# Carnosic Acid Directly Targets STING C‐Terminal Tail to Improve STING‐Mediated Inflammatory Diseases

**DOI:** 10.1002/advs.202417686

**Published:** 2025-02-18

**Authors:** Wenqing Mu, Guang Xu, Ling Li, Jincai Wen, Ye Xiu, Jia Zhao, Tingting Liu, Ziying Wei, Wei Luo, Huijie Yang, Zhixin Wu, Xiaoyan Zhan, Xiaohe Xiao, Zhaofang Bai

**Affiliations:** ^1^ Department of Hepatology the Fifth Medical Center of PLA General Hospital Beijing 100039 China; ^2^ School of Traditional Chinese Medicine Capital Medical University Beijing 100069 China; ^3^ State Key Laboratory of Radiation Medicine and Protection Institutes for Translational Medicine Soochow University Jiangsu 215123 China; ^4^ Beijing Institute of Biotechnology Beijing 100071 China; ^5^ Military Institute of Chinese Materia Fifth Medical Center of Chinese PLA General Hospital Beijing 100039 China

**Keywords:** carnosic acid, cGAS‐STING, inflammatory responses, STING inhibitors, Trex1 deficiency

## Abstract

cGAS (cyclic GMP‐AMP synthase)‐STING (stimulator of interferon genes) signaling plays a vital role in innate immunity, while its deregulation may lead to a wide variety of autoinflammatory and autoimmune diseases. It is essential to identify specifically effective lead compounds to inhibit the signaling. Herein, it is shown that carnosic acid (CA), an active ingredient of medicinal plant *Rosmarinus officinalis L*., specifically suppressed cGAS‐STING pathway activation and the subsequent inflammatory responses. Mechanistically, CA directly bound to STING C‐terminal tail (CTT), impeded the recruitment of TANK‐binding kinase 1 (TBK1) onto STING signalosome, thereby blocking the phosphorylation of STING and interferon regulatory factor 3 (IRF3) nuclear translocation. Importantly, CA dramatically attenuated STING‐mediated inflammatory responses in vivo. Consistently, CA has a salient ameliorative effect on autoinflammatory disease model mediated by *Trex1* deficiency, via inhibition of the cGAS‐STING signaling. Notably, the study further indicates that phenolic hydroxyl groups are essential for CA‐mediated STING inhibitory activity. Collectively, the results thus identify STING as one of the crucial targets of CA for mediating CA's anti‐inflammatory activity, and further reveal that STING CTT may be a novel promising target for drug development.

## Graphical abstract

This study presents carnosic acid (CA), an active ingredient of *Rosmarinus officinalis L*., dramatically ameliorates *Trex1* deficiency‐mediated and STING‐dependent systemic inflammatory diseases by targeting STING C‐terminal tail (CTT) to disrupt STING activity. The study provides a potential drug candidate for the treatment of STING‐driven inflammatory diseases and indicates that STING CTT is a promising drug target.

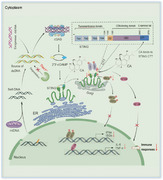

## 1 Introduction

cGAS (cyclic GMP‐AMP synthase)‐STING (stimulator of interferon genes) pathway mediates powerful innate immune defense programs with unique mechanisms. Within this signaling pathway, the identification as well as binding of cGAS to exogenous or endogenous cytosolic double‐stranded DNA (dsDNA) catalyzes the generation of 2′3′‐cGAMP, a second messenger molecule and potent agonist of endoplasmic reticulum (ER) membrane protein STING.^[^ ^1^, ^2^, ^3^ ^]^ After binding to cGAMP, STING undergoes extensive conformational rearrangement and subsequently drives itself to translocation from the ER to Golgi/ER‐Golgi intermediate compartment (ERGIC). On reaching the Golgi or ERGIC, STING recruits TANK‐binding kinase 1 (TBK1) and promotes autophosphorylation of TBK1 and phosphorylation of STING to allow for the recruitment of interferon regulatory factor 3 (IRF3).^[^ ^3^, ^4^ ^]^ Moreover, TBK1‐induced IRF3 phosphorylation enables nuclear translocation of IRF3, which triggers gene expression of interferons (IFNs), IFN‐related genes, and other inflammatory mediators.^[^ ^4^ ^]^

The appropriate activation of cGAS‐STING pathway helps host defend against bacterial and viral invasion. Recent study has shown that mice with *Sting* deficiency are more susceptible to infection by virus.^[^ ^5^ ^]^ Furthermore, numerous studies also identified the pivotal role of cGAS‐STING signaling in anti‐tumor immunity and some targeted anti‐tumor drugs have been identified.^[^ ^6^, ^7^, ^8^ ^]^ However, as a double‐edged sword, the aberrant activation of cGAS‐STING results in overexpression of IFNs and inflammatory cytokines, which have been implicated in the development of autoimmune and autoinflammatory diseases. For example, Aicardi‐Goutières syndrome (AGS) is an autoinflammatory disorder caused by mutations in genes including DNAse *Trex1*, *RNASEH2A/B/C*, or *Samhd1*, which is associated with the disorder of self‐DNA metabolism and activation of cGAS‐STING signaling.^[^ ^9^, ^10^ ^]^ Furthermore, it has been reported that pathology due to defects in *AGS* gene subset is improved in *cGAS* or *Sting‐deficient* (*Sting^−/−^*) mice.^[^ ^11^ ^]^ Mutations in *Sting* can cause STING‐associated vasculopathy with onset in infancy (SAVI), a severe autoinflammatory syndrome in children.^[^ ^12^ ^]^ Moreover, elevated levels of cGAMP have been detected in serum from patients with systemic lupus erythematosus (SLE), this is strong evidence of the presence of cGAS‐STING pathway activity.^[^ ^13^ ^]^ Additionally, blocking cGAS‐STING signaling can improve multiple IFN or inflammation‐driven diseases, such as rheumatoid arthritis,^[^ ^14^ ^]^ non‐alcoholic fatty liver disease,^[^ ^15^ ^]^ sepsis,^[^ ^16^ ^]^ aging,^[^ ^17^ ^]^ psoriasis,^[^ ^18^ ^]^ and Parkinson disease.^[^ ^19^ ^]^ In light of this, cGAS‐STING has emerged as an attractive drug target in the treatment of autoimmune or autoinflammatory disorders. Notably, clinical drugs targeting the cGAS‐STING pathway are still not available. Although several cGAS‐STING pathway inhibitors have been identified,^[^ ^4^, ^20^, ^21^, ^22^ ^]^ they have limited potential for clinical applications. Thus, it is urgent to develop cGAS‐STING inhibitors with high safety for clinical trials.

*Rosmarinus officinalis L*. is well‐known for its wide range of medicinal and edible values. Previous studies have assessed its considerable antioxidant and anti‐inflammatory activity for therapeutic potential in diseases such as arthritis,^[^ ^23^ ^]^ fibrosis, ^[^ ^24^ ^]^ and neuroinflammation.^[^ ^25^ ^]^ As the major active ingredient of *Rosmarinus officinalis L*., the therapeutic effects of carnosic acid (CA) have been identified in multiple diseases. For example, Recent studies showed that CA protects against Doxorubicin‐mediated cardiotoxicity by activating nuclear factor erythroid 2‐related factor (Nrf2) to inhibit oxidative stress, apoptosis, and pyroptosis.^[^ ^26^ ^]^ Moreover, CA can also alleviate lipopolysaccharide‐induced systemic inflammation and monosodium urate (MSU)‐induced peritonitis by blocking the assembly and activation of NLRP3 inflammasome.^[^ ^27^ ^]^ Additionally, previous docking studies demonstrated that CA and carnosol alleviate inflammatory pain by inhibiting microsomal prostaglandin E2 synthase‐1 (mPGES‐1) and 5‐lipoxygenase (5‐LO) activity.^[^ ^28^ ^]^ Although the anti‐inflammatory activity of CA is emerging, knowledge of its therapeutic potential in other diseases, as well as its biological activity, remains limited.

In this study, we have identified the ability of CA to antagonize STING activity. Our results suggested that CA directly bound to the STING C‐terminal tail (CTT), impeded the recruitment of TBK1 onto STING signalosome, thereby blocking the phosphorylation of STING and IRF3. Moreover, CA dramatically attenuated STING‐mediated inflammatory responses in vivo. Importantly, CA strongly alleviated autoinflammatory responses triggered by *Trex1* deficiency. Interestingly, we also found that the phenolic hydroxyl groups are essential for CA to exert STING inhibitory activity, as its inhibitory effect was disrupted once the phenolic hydroxyl groups of CA were replaced by other groups. Accordingly, our findings identify and characterize a natural STING‐specific antagonist, which may be a promising strategy for developing drugs to treat STING‐mediated autoinflammatory or autoimmune diseases.

## 2 Results

### 2.1 CA Selectively Suppresses Cytosolic DNA‐Triggered cGAS‐STING Pathway Activation and Downstream Gene Expression

**Figure 1:**
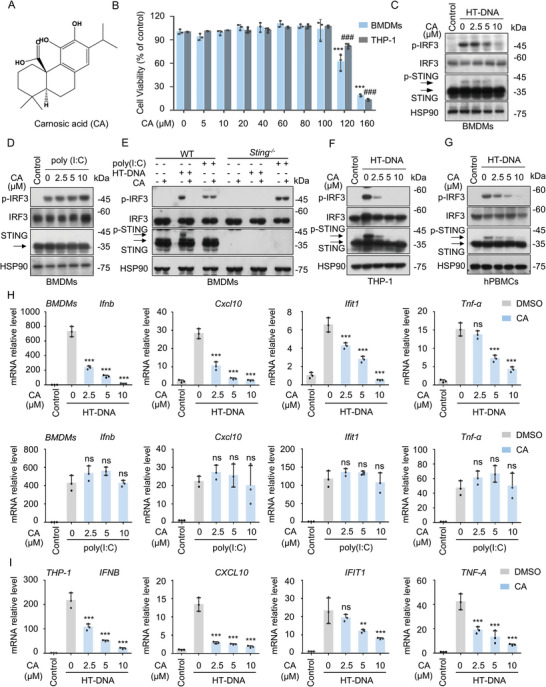
CA Selectively Blocks the Cytoplasmic DNA‐Triggered cGAS‐STING Signaling Activation and Downstream Gene Expression. A) Chemical structure of CA. B) Cell viability of BMDMs and THP‐1 cells administrated with the indicated concentrations of CA for 12 h. C,D) BMDMs were administrated with vehicle control (DMSO, 1000x) or the indicated concentrations of CA for 1 h followed by stimulation with HT‐DNA (2 µg mL^−1^) (C) or poly (I:C) (2 µg mL^−1^) (D) for 2 h, respectively. Phosphorylation of IRF3 (p‐IRF3) and STING (p‐STING) were evaluated by immunoblot assay. E) WT BMDMs and *Sting^−/−^* BMDMs were pretreated with vehicle control (DMSO) or CA (10 µM) for 1 h followed by stimulation with HT‐DNA (2 µg mL^−1^) or poly (I:C) (2 µg mL^−1^) for 2 h, respectively. The expression of p‐IRF3 and p‐STING were assessed by western blot. F, G) PMA‐primed THP‐1 cells (F) and hPBMCs (G) were pretreated with the indicated concentrations of CA for 1 h, and then stimulated with HT‐DNA for 2 h. The expression of p‐IRF3 and p‐STING were detected by western blot. H) BMDMs were pretreated with the indicated concentrations of CA for 1 h and then treated with HT‐DNA and poly (I:C) for 4 h, respectively. Induction of *Cxcl10*, *Ifnb*, *Ifit1*, and *Tnf‐α* were measured by qPCR assay. I) PMA‐primed THP‐1 cells were pretreated with the indicated concentrations of CA and then stimulated with HT‐DNA. Induction of *IFNB*, *CXCL10*, *IFIT1*, and *TNF‐α* were detected by qPCR assay. Data are presented as mean ± s.d. (n = 3). ^**^p < 0.01, ^***^p < 0.001, ^###^ *p* < 0.001; ns, no significance. Gene expression was normalized to Actin. One‐Way ANOVA followed by the Dunnett's post hoc test.

To investigate the effect of CA (**Figure** **1**A) on cGAS‐STING pathway activation, we first examined the cytotoxicity of CA. As shown in Figure 1B, no significant decrease in cell viability was observed after cells were treated with 100 µM CA for 12 h. Next, we assessed the role of CA in the activation of cGAS‐STING signaling triggered by herring testis DNA (HT‐DNA).^[^ ^29^ ^]^ The results showed that CA dramatically blocked HT‐DNA‐triggered phosphorylation of STING and IRF3 (Figure 1C; Figure S1A,B, Supporting Information) in a concentration‐dependent manner. Given that IRF3 was also regulated by RNA‐sensing retinoic‐acid‐inducible gene‐I (RIG‐I)‐mitochondrial antiviral‐signaling protein (MAVS) signaling, we subsequently assessed the effect of CA on the activation of the RIG‐I pathway induced by cytosolic RNA mimics poly (I:C).^[^ ^30^ ^]^ Notably, CA had no effect on poly (I:C)‐induced phosphorylation of IRF3 (Figure 1D; Figure S1C, Supporting Information). To further determine the specificity of CA for regulation of the cGAS‐STING pathway. We compared the effect of CA on HT‐DNA and poly(I:C)‐induced IRF3 phosphorylation in the presence or absence of *Sting*. As expected, CA did not affect poly (I:C)‐triggered activation of the RIG‐I pathway in either wild type (WT) bone marrow‐derived macrophages (BMDMs) or *Sting^−/−^* BMDMs (Figure 1E; Figure S1D,E, Supporting Information). Moreover, we further investigated the effect of CA on stimulation of other innate immune receptor ligands, such as DNA mimics poly (dA:dT) and interferon stimulatory DNA (ISD), STING agonist 10‐carboxymethyl‐9‐acridanone (CMA), and TLR ligand lipopolysaccharide (LPS). Notably, CA treatment potently inhibited IRF3 phosphorylation induced by the cGAS‐STING pathway agonists poly (dA:dT), ISD, and CMA, but had no effect on IRF3 phosphorylation induced by TLR ligand LPS (Figure S1F,G, Supporting Information). Similarly, in THP‐1 and human peripheral blood mononuclear cells (hPBMCs), CA treatment dramatically inhibited the phosphorylation of STING and IRF3 induced by HT‐DNA (Figure 1F,G; Figure S1H–K, Supporting Information).

Consistent with these immunoblotting results, CA apparently repressed the IRF3‐responsive genes (*Cxcl10*, *Ifnb*, and *Ifit1*) and NF‐κB‐responsive genes (*Tnf‐α* and *Il‐6*) expression triggered by HT‐DNA (Figure 1H; Figure S2A, Supporting Information), whereas CA did not affect the expression of these genes triggered by poly (I:C) (Figure 1H; Figure S2B, Supporting Information). Similarly, CA impaired the induction of *CXCL10*, *IFNB*, *IFIT1*, *TNF‐α*, *ISG15*, and *IL‐6* mRNA stimulated by HT‐DNA in human THP‐1 cells (Figure 1I; Figure S2C,D, Supporting Information). Additionally, CA potently inhibited the mRNA levels of *Ifnb*, *Cxcl10*, and *Tnf‐α* induced by ISD, poly (dA:dT) and CMA, but had no effect on the mRNA levels of these genes regulated by LPS (Figure S2E–G, Supporting Information). Taken together, these results reveal that CA selectively attenuates the cGAS–STING pathway, with no effect on RIG‐I and TLR‐dependent pathways.

### 2.2 CA Directly Impairs STING Signalosome Activation

**Figure 2:**
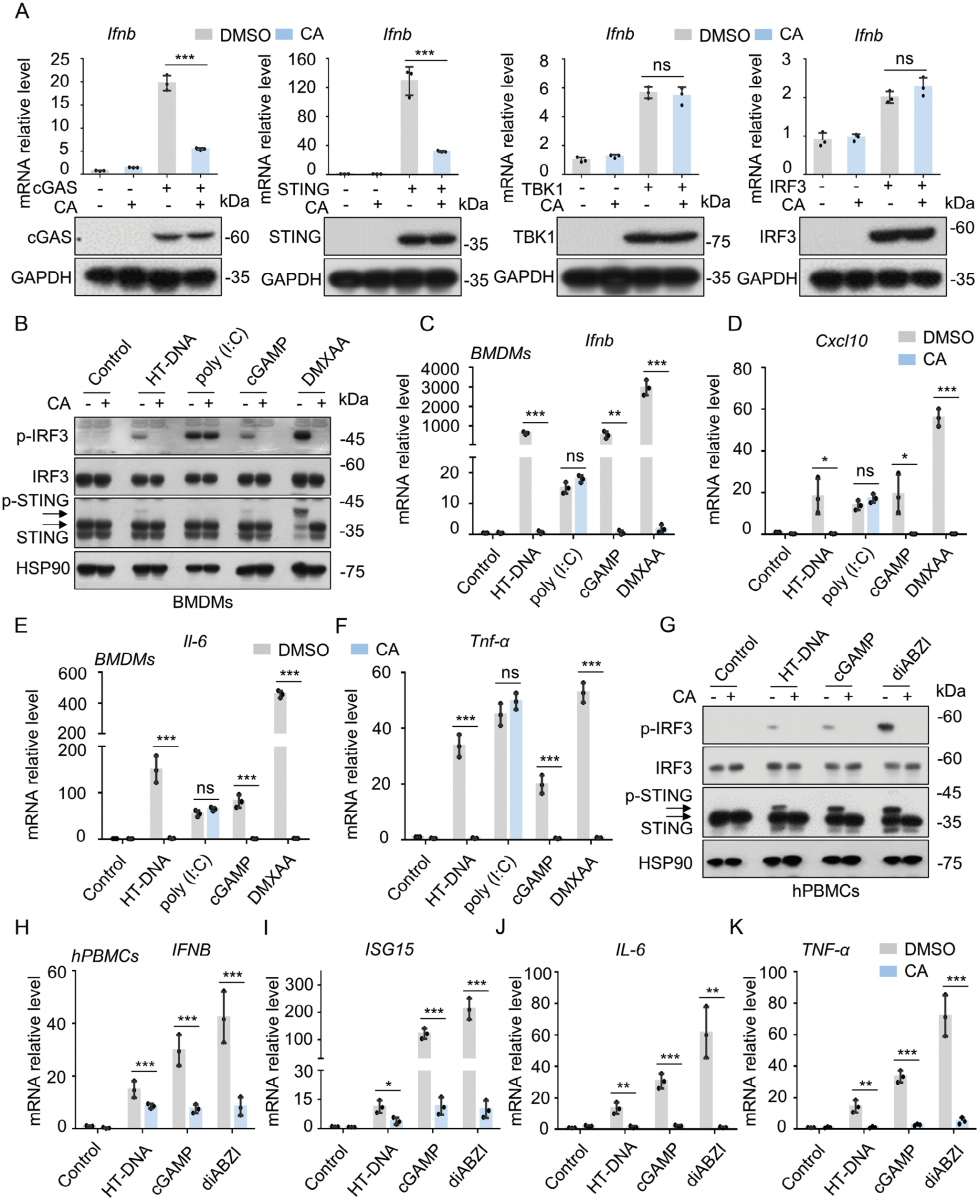
CA Directly Inhibits STING Signalosome Activation. A) HEK‐293 cells were transfected with their vector or Flag‐tagged cGAS, STING, IRF3, and TBK1 for 18 h followed by administration with vehicle control (DMSO, 1000x) or CA (10 µM) for 6 h. Induction of *IFNB* was analyzed by qPCR assay. Immunoblot detected the expression of Flag‐tagged cGAS, STING, TBK1, and IRF3. B) Immunoblot assays of p‐IRF3 and p‐STING from BMDMs pretreated with vehicle control (DMSO) or CA (10 µM) for 1 h followed by stimulation with HT‐DNA (2 µg mL^−1^), poly (I:C) (2 µg mL^−1^), cGAMP (2 µg mL^−1^), or DMXAA (15 µg mL^−1^) for 2 h. C‐F) qPCR analysis of *Ifnb* (C), *Cxcl10* (D), *Ifit1* (E), and *Il‐6* (F) from BMDMs pretreated with CA (10 µM) for 1 hour followed by treatment with HT‐DNA, poly (I:C), cGAMP, or DMXAA for 4 h. G) Immunoblot assays of p‐IRF3 and p‐STING from hPBMCs administrated with CA (10 µM) for 1 h followed by treatment with HT‐DNA, cGAMP, or diABZI for 2 h. H‐K) qPCR analysis of *IFNB* (H), *ISG15* (I), *IL‐6* (J), and *TNF‐α* (K) mRNA levels from hPBMCs pretreated with CA (10 µM) for 1 h followed by treatment with HT‐DNA, cGAMP, or diABZI for 4 h. Data are displayed as mean ± s.d. (n = 3). ^*^p < 0.05, ^**^p < 0.01, ^***^p < 0.001; statistics differences were analyzed using an unpaired Student's *t*‐test. Gene expression was normalized to Actin.

The adaptors in cGAS‐STING pathway were screened to identify the CA target protein. HEK293 cells were transfected with Flag‐tagged cGAS, STING, IRF3, and TBK1, respectively, and then treated with CA. The induction of *IFNB* mRNA was detected. The results showed that CA dramatically decreased the *IFNB* mRNA levels induced by cGAS and STING, but had no influence on TBK1 or IRF3‐triggered *IFNB* mRNA expression (**Figure** **2**A), suggesting that CA restricted the cGAS‐STING pathway by targeting STING signalosome or its upstream. Moreover, CA remarkably inhibited the phosphorylation of IRF3, TBK1 and STING mediated by STING overexpression (Figure S3A, Supporting Information), whereas it had no effect on the phosphorylation of IRF3 and TBK1 induced by TBK1 overexpression (Figure S3B, Supporting Information). Similarly, phosphorylation of IRF3 itself after overexpression was not inhibited by CA (Figure S3C, Supporting Information).

To substantiate these findings, we assessed the effect of CA on STING signalosome activation triggered by endogenous STING ligand cGAMP (2′3′‐cGAMP) and 5,6‐dimethylxanthenone‐4‐acetic acid (DMXAA).^[^ ^31^ ^]^ CA treatment impaired the phosphorylation of STING and IRF3 triggered by HT‐DNA, cGAMP, and DMXAA (Figure 2B; Figure S3D,E, Supporting Information). Moreover, CA apparently inhibited IRF3‐responsive genes (*Ifnb*, *Cxcl10*, and *Ifit1*) and NF‐κB‐responsive genes (*Tnf‐α* and *Il‐6*) expression stimulated by them (Figure 2C–F; Figure S3F, Supporting Information). Similarly, CA treatment also blocked the HT‐DNA, diABZI,^[^ ^32^ ^]^ and cGAMP‐triggered STING signalosome activation (Figure 2G; Figure S3G,H, Supporting Information) and relative genes expression in hPBMCs (Figure 2H–K; Figure S3I,J, Supporting Information).

**Figure 3:**
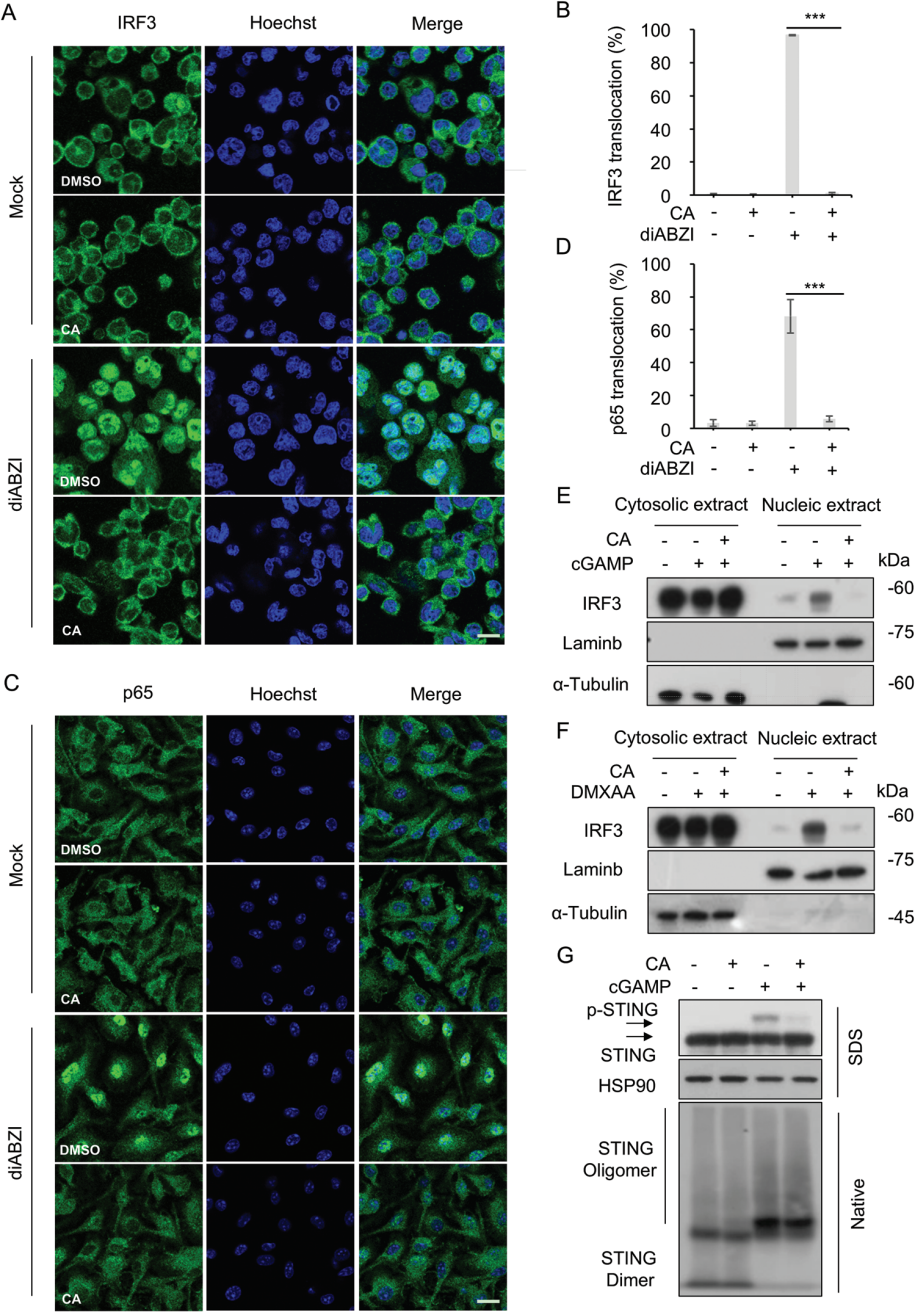
CA Blocks Nuclear Translocation of p65 and IRF3. A) Immunofluorescence analysis of nuclear translocation of IRF3 from THP‐1 cells pretreated with vehicle control (DMSO, 1000x) or CA (10 µM) for 1 h followed by administration with diABZI (10 µg mL^−1^) for 2 h. Scale bars, 25 µm. B) Quantification of cells with nuclear IRF3. C) Immunofluorescence analysis of nuclear translocation of p65 from BMDMs pretreated with CA (10 µM) for 1 h followed by stimulation with diABZI (10 µg mL^−1^) for 2 h. Scale bars, 25 µm. D) Quantification of cells with nuclear p65. E, F) Immunoblot assays of cytosolic and nucleic indicated proteins from BMDMs administrated with CA (10 µM) for 1 h followed by stimulation with cGAMP (2 µg mL^−1^) (E) or DMXAA (15 µg mL^−1^) (F) for 2 h. G) Western blot assays of STING oligomerization from BMDMs treated with CA (10 µM) for 1 h followed by stimulation with cGAMP for 2 h. Data are shown as mean ± s.d. (n = 3). ***p < 0.001. One‐Way ANOVA followed by the Dunnett's post hoc test.

Additionally, our further investigated showed that CA dramatically hampered the nuclear translocation of IRF3 triggered by diABZI (**Figure** **3**A,B), as well as cGAMP (Figure 3E) or DMXAA (Figure 3F). The inhibition of CA on diABZI‐induced the nuclear translocation of p65 was also observed (Figure 3C,D). Notably, CA treatment decreased the phosphorylation of STING but did not affect its oligomerization induced by cGAMP (Figure 3G), suggesting that CA may be able to exert inhibitory activity by acting downstream of STING oligomerization. Taken together, our results demonstrate a direct regulatory effect of CA on STING signalosome.

### 2.3 CA Directly Binds to STING C‐terminal Tail

**Figure 4:**
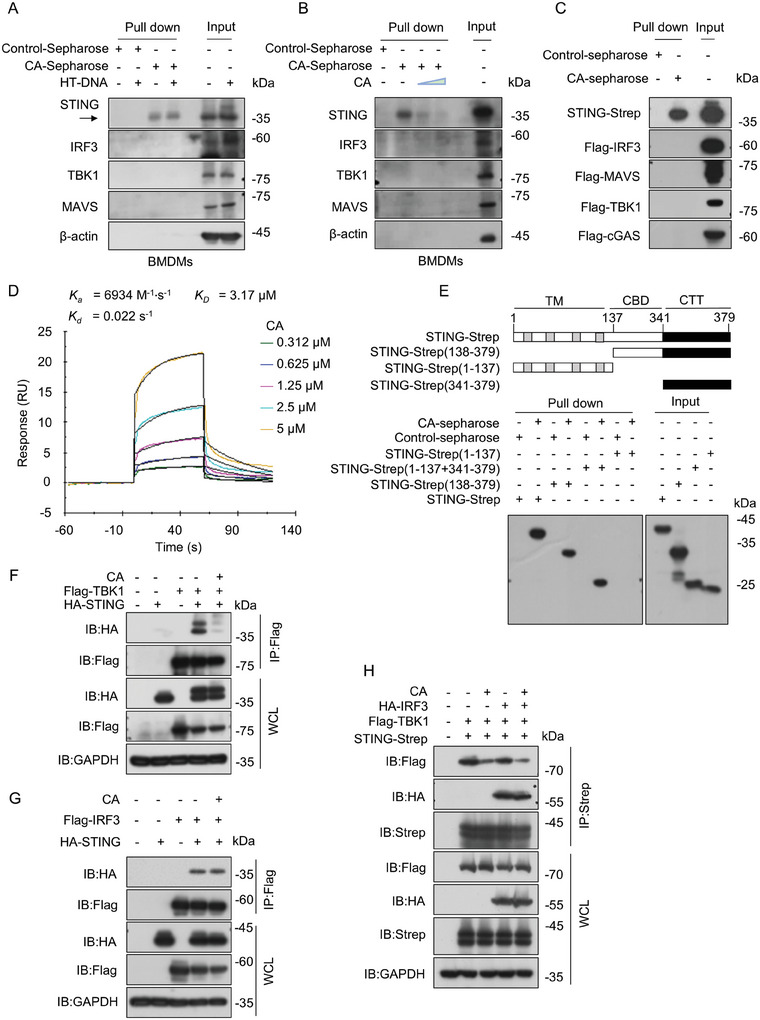
CA Targets the CTT Binding Domain of STING and Competitive Impairs the Recruitment of TBK1 onto STING Signalosome. A) Cell lysates of BMDMs with or without HT‐DNA (2 µg mL^−1^) stimulation incubated with control‐sepharose or CA‐sepharose. B) Cell lysates from BMDMs incubated with control‐sepharose, CA‐sepharose in the presence or absence of free CA (0.2 mM and 0.4 mM). C) HEK293T cells were transfected with indicated plasmids or their vector for 24 hours. Next, lysed cells were harvested and incubated with control‐sepharose or CA‐sepharose. D) The kinetics of CA binding to STING were assessed by SPR assay. E) Schematic of STING as well as its truncation mutants (top). HEK293T cells were transfected with their vector or Strep‐tagged STING and its mutants (1‐137, 1–137+341‐379, or 138–379) for 24 h, respectively. Then cell lysates were harvested and incubated with control‐sepharose or CA‐sepharose. (A‐C and E) The pull‐down samples were analyzed using immunoblot. F, G) HEK293T cells were co‐transfected with their vector or Flag‐tagged TBK1 (F) (or Flag‐tagged IRF3 (G)) and HA‐tagged STING for 18 h followed by treatment with vehicle control (DMSO, 1000x) or CA (20 µM) for 6 h. The cell lysates were immunoprecipitated with Flag‐tagged beads and assessed using western blot. H) HEK293T cells were co‐transfected with Strep‐tagged STING, HA‐tagged IRF3, and Flag‐tagged TBK1 for 18 h followed by treatment with vehicle control (DMSO) or CA (20 µM) for 6 h. The cell lysates were immunoprecipitated with Strep‐tagged beads and assessed using western blot.

To determine the protein target of CA in cGAS‐STING pathway, a pull‐down assay was performed. CA‐conjugated epoxy activated sepharose (CA‐sepharose) was employed to incubate with cell lysates from BMDMs stimulated with HT‐DNA or not, and the pulled‐down protein was detected. Notably, only STING was precipitated by CA‐sepharose, while IRF3, TBK1, and MAVS were not (**Figure** **4**A). Moreover, we also performed a competitive assay to further identify the specificity of the interaction between CA and STING. The results showed that the pull‐down of STING was competed off by free CA (Figure 4B). Additionally, Strep‐tagged STING and Flag‐tagged IRF3, TBK1, cGAS, and MAVS were overexpressed in HEK293T cells. Consistent with the above findings, pull‐down assay also presented that only Strep‐tagged STING was precipitated by CA‐sepharose (Figure 4C). To further determine the binding of STING to CA, surface plasmon resonance (SPR) was performed to measure the binding affinity and kinetics between CA and STING proteins. The results showed that soluble human STING bound CA with an affinity (*K_D_*) of 3.17 µM (Figure 4D). In addition, we also quantified the binding affinity between CA and STING by microscale thermophoresis (MST) assay. As shown in Figure S4A (Supporting Information), The *K_D_* value between CA and STING was 10 µM. Collectively, these data suggest that CA can directly bind to STING protein.

To map the CA‐binding region of STING, full‐length STING and STING truncation mutants (1‐137**—**TM domain, 138‐379**—**CTD domain, and 1–137+341‐379**—**TM + CTT domain) were overexpressed in HEK293T cells, respectively. Next, the pull‐down assay was performed to detect pull‐down protein. The results showed that CA targets STING C‐terminal tail (CTT, 341–379)(Figure 4E; Figure S4B, Supporting Information).

### 2.4 CA Blocks the Recruitment of TBK1 onto STING Signalosome

Considering that the C‐terminal tail of STING is involved in the interaction with TBK1 and IRF3 recruitment.^[^ ^33^, ^34^ ^]^ We further evaluated the influence of CA on the recruitment of TBK1 or IRF3 onto STING CTT. Interestingly, we noticed that CA conspicuously weakened the interaction between STING and TBK1 but did not affect the recruitment of IRF3 to STING (Figure 4F,G). To further determine whether CA influences the assembly of the STING‐TBK1‐IRF3 complex, STING, TBK1, and IRF3 were co‐transfected into HEK293T cells followed by CA treatment. CA only impaired the interaction between STING and TBK1 without affecting the interaction between IRF3 and STING (Figure 4H). These data suggested that CA directly binds to STING CTT and impaired the recruitment of TBK1 onto STING signalosome.

### 2.5 Phenolic Hydroxyl Groups are Essential for CA‐Mediated STING Inhibitory Activity

**Figure 5:**
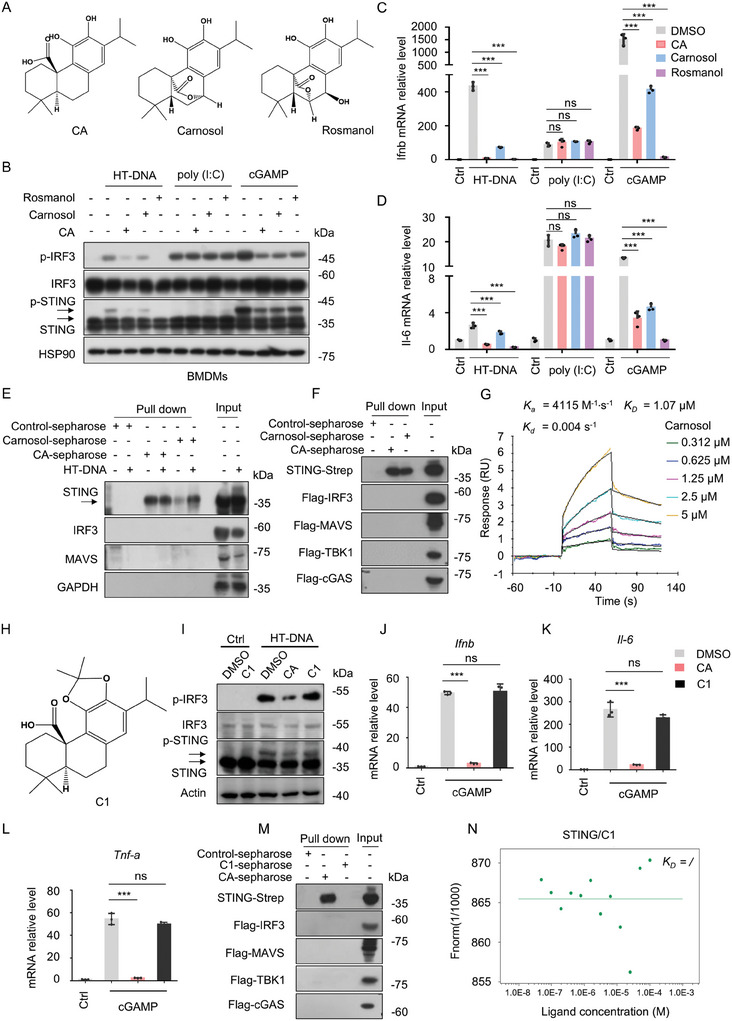
The Phenolic Hydroxyl Groups Are Essential for CA‐Mediated STING Inhibitory Activity. A) Chemical structures of CA and its derivatives carnosol and rosmanol. B) BMDMs were pretreated with vehicle control (DMSO, 1000x) or CA (10 µM), carnosol (10 µM), and rosmanol (10 µM) for 1 h followed by stimulation with HT‐DNA (2 µg mL^−1^), poly (I:C) (2 µg mL^−1^), or cGAMP (2 µg mL^−1^) for 2 h. The p‐IRF3 and p‐STING were evaluated by western blot. C,D) qPCR analysis of *Ifnb* (C) and *Il‐6* (D) from BMDMs administrated with CA (10 µM), carnosol (10 µM), or rosmanol (10 µM) for 1 hour followed by poly (I:C), HT‐DNA, or cGAMP stimulation for 4 h. E) Cell lysates from BMDMs with or without HT‐DNA stimulation incubated with control‐sepharose, carnosol‐sepharose, or CA‐sepharose. F) HEK293T cells were transfected with indicated plasmids for 24 h. Next, lysed cells were harvested and then incubated with control‐sepharose, carnosol‐sepharose, or CA‐sepharose, respectively. (E and F) The pull‐down samples were analyzed by immunoblot. G) Binding affinity of carnosol with human STING was determined using an SPR assay. RU, response units. H) Chemical structure of C1. I) BMDMs were treated with vehicle control (DMSO) or CA (10 µM) and its derivatives C1 (10 µM) for 1 h followed by stimulation with HT‐DNA (2 µg mL^−1^). The p‐IRF3 and p‐STING were evaluated by western blot. J‐L) BMDMs were treated with CA (10 µM) or C1 (10 µM) for 1 h followed by stimulation with cGAMP (2 µg mL^−1^) for 4 h. The induction of *Ifnb* (J), *Il‐6* (K), and *Tnf‐a* (L) mRNA levels were assessed by qPCR assay. M) HEK293T cells were transfected with indicated plasmids for 24 h. The lysed cells were harvested and incubated with C1‐sepharose or CA‐sepharose, respectively. The pull‐down samples were analyzed by immunoblot. N) Binding affinity of C1 with human STING was determined using MST assay. Data were expressed as the mean ± s.d. (n = 3); ***p < 0.001; ns, no significance; One‐Way ANOVA followed by the Dunnett's post hoc test.

Previous studies identified SN‐011, an inhibitor of STING binding pocket that contains a phenolic hydroxyl group. A structure–activity relationship (SAR) study revealed that the phenolic hydroxyl group played an important role in the inhibition of STING activation by SN‐011.^[^ ^21^ ^]^ To assess the role of the phenolic hydroxyl groups in the inhibition of STING activation by CA, we first screened out two derivatives with a similar structure to CA, carnosol, and rosmanol, which only substituted the carboxyl group in CA (**Figure** **5**A). Consistent with the inhibitory activity of CA, both carnosol and rosmanol selectively blocked HT‐DNA and cGAMP‐induced activation of cGAS‐STING pathway (Figure 5B; Figure S5A,B, Supporting Information). As expected, carnosol and rosmanol also apparently impaired the induction of downstream genes (*Ifnb*, *Cxcl10*, *Isg15*, and *Il‐6*) triggered by HT‐DNA and cGAMP, whereas they did not influence these genes expression stimulated by poly(I:C) (Figure 5C,D; Figure S5C,D, Supporting Information). Moreover, pull‐down assay showed that only STING protein was precipitated by carnosol‐sepharose, whereas IRF3, TBK1, cGAS, and MAVS did not bind to carnosol (Figure 5E,F). Additionally, we also quantified the affinity of carnosol to STING by SPR assay. The results showed that the STING bound carnosol with an affinity (*K_D_*) of 1.07 µM (Figure 5G).

To further elucidate the critical role of the phenolic hydroxyl groups in the STING inhibitory activity of CA, we synthesized a CA derivative compound 1 (C1) without phenolic hydroxyl groups (Figure 5H). As shown in Figure 5I and Figure S5I (Supporting Information), CA but not its derivative C1 significantly blocked HT‐DNA and cGAMP‐induced activation of the cGAS‐STING pathway. Consistent with the results of immunoblotting, C1 did not affect the mRNA levels of HT‐DNA and cGAMP‐induced downstream response factors (Figure 5J–L; Figure S5E–H, Supporting Information). Moreover, further pull‐down assay showed that CA derivative C1 did not bind STING (Figure 5M). Subsequently, MST assay was performed to quantify the binding affinity between C1 and STING. Consistent with the pull‐down assay, the results showed that C1 did not display any affinity to STING (Figure 5N). Overall, our results demonstrate that the phenolic hydroxyl groups are essential for the inhibitory activity of STING mediated by CA.

### 2.6 CA Attenuates STING‐Mediated Inflammatory Responses In Vivo

**Figure 6:**
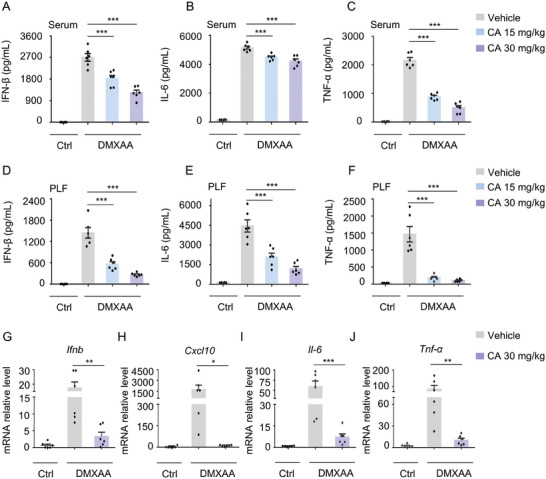
CA Dramatically Attenuates STING‐Mediated Inflammatory Responses In Vivo. A‐F) Female C57/BL6 mice were administrated with vehicle control or CA (15 mg kg^−1^ and 30 mg kg^−1^) for 2 h followed by treatment with vehicle control or DMXAA (30 mg kg^−1^) for 5 h. ELSA analysis of IFN‐β (A), IL‐6 (B), and TNF‐α (C) in mouse serum as well as IFN‐β (D), IL‐6 (E), and TNF‐α (F) in mouse PLF. G‐J) qPCR analysis of *Ifnb* (G), *Cxcl10* (H), *Il‐6* (I), and *Tnf‐α* (J) mRNA expression from mouse PLF pretreated with CA (30 mg kg^−1^) for 2 h followed by treatment with DMXAA (30 mg kg^−1^) for 5 h. Data are displayed as mean ± s.e.m. (n = 6). ^*^p < 0.05, ^**^p < 0.01, ^***^p < 0.001. Gene expression was normalized to Actin. One‐Way ANOVA followed by the Dunnett's post hoc test.

The most basic and prominent feature of autoinflammatory type I interferonopathies is the aberrant/persistent generation and activation of type I IFN. In the following in vivo experiment, we further assessed the physiological relevance of CA function in the context of aberrant activation of STING. The results showed that mice pretreatment with DMXAA induced severe inflammatory responses with high production of IFN‐β, TNF‐α, as well as IL‐6, while mice administered CA resisted the inflammation in a dose‐dependent manner, displaying an attenuated production of IFN‐β, TNF‐α, and IL‐6 in both mice serum (**Figure** **6**A–C) and peritoneal lavage fluid (PLF) (Figure 6D–F). Furthermore, qPCR analysis showed that CA apparently repressed the inflammatory factors mRNA levels in mouse PLF, such as *Ifnb*, *Cxcl10*, *Tnf‐α*, and *Il‐6* (Figure 6G–J). Collectively, these findings declare that CA dampens the generation of type I IFN and proinflammatory cytokines in vivo, which may be achieved by blocking the aberrant activation of STING signaling.

### 2.7 CA Attenuates the Autoinflammatory Responses in *Trex1* Deficiency Mice

**Figure 7:**
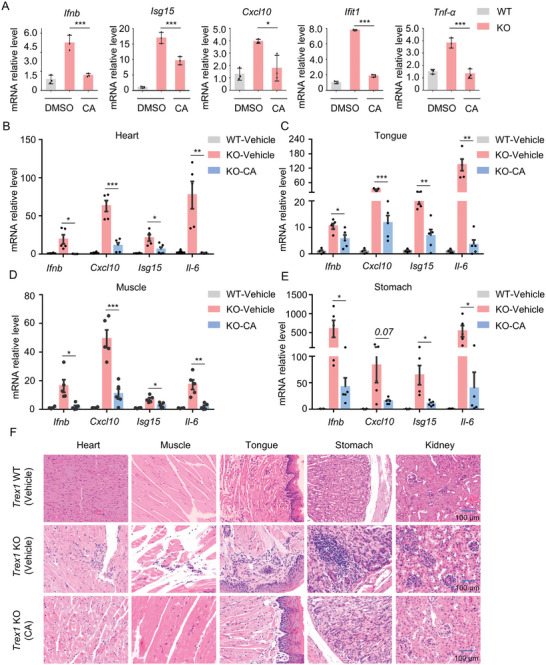
CA Alleviates Systemic Inflammation in *Trex1* Deficiency Mice. A) qPCR analysis of *Ifnb*, *Isg15*, *Cxcl10*, *Ifit1*, and *Tnf‐α* mRNA levels from *Trex1^−/−^* BMDMs treated with DMSO or CA (10 µM) for 10 h. Data are presented as mean ± s.d. (n = 3); ^*^p < 0.05, ^***^p < 0.001. Gene expression was normalized to Actin. One‐Way ANOVA followed by the Dunnett's post hoc test. B‐E) qPCR analysis of *Cxcl10*, *Ifnb*, *Il‐6*, and *Isg15* mRNA levels from mouse heart (B), tongue (C), muscle (D), as well as stomach (E) tissues, which were injected intraperitoneally with vehicle control or CA (20 mg kg^−1^) for 14 consecutive days. Data are presented as mean ± s.e.m. (n = 4 WT group; n = 5 *Trex1^−/−^* groups). ^*^p < 0.05, ^**^p < 0.01, ^***^p < 0.001. ns, no significance. Gene expression was normalized to Actin. Statistics differences were analyzed using an unpaired Student's *t*‐test. F) Representative H&E‐stained tissue sections (muscle, heart, tongue, stomach, and kidney) from *Trex1^−/−^* or WT mice with vehicle control or CA (20 mg kg^−1^) for 14 consecutive days. Images in (F) are 20x magnification.

Considering that the overactivation of STING‐dependent signaling mediated by aberrant cytosolic DNAs may lead to serious autoimmune or autoinflammatory diseases, we inferred whether CA could be used therapeutically to abate the STING‐mediated autoinflammatory disorders. It has been reported that the autoinflammatory disease in *Trex1‐deficient* (*Trex1^−/−^*) mice was mechanistically related to STING‐dependent signaling.^[^ ^35^, ^36^ ^]^ Thus, we first monitored the influence of CA on the mRNA expression of type I IFN and related inflammatory cytokines in *Trex1^−/−^* BMDMs. As expected, CA suppressed *Ifnb*, *Isg15*, *Cxcl10*, *Ifit1*, and *Tnf‐α* expression in *Trex1^−/−^* BMDMs (**Figure** **7**A). We also examined these genes levels in different tissues (muscle, heart, tongue, and stomach) of *Trex1^−/−^* or WT mice with CA treatment or not. Notably, CA treatment consistently inhibited *Ifnb*, *Cxcl10*, *Tnf‐α*, and *Il‐6* mRNA levels in heart, tongue, muscle, and stomach of *Trex1^−/−^* mice (Figure 7B–E). Moreover, hematoxylin and eosin (H&E) staining displayed that CA attenuated the inflammatory symptoms in muscle, heart, stomach, tongue, and kidney tissues of *Trex1^−/−^* mice (Figure 7F). In summary, these data suggest that CA could improve STING‐mediated autoimmune or autoinflammatory disease.

### 2.8 CA Exhibits High Safety and Selectivity Compared to H‐151

Considering the potential of CA in ameliorating autoinflammatory diseases mediated by aberrant activation of STING. We compared the inhibitory effects of CA with STING palmitoylation inhibitor H‐151 on STING‐mediated inflammatory response in vivo. The results showed that CA and H‐151 pretreatment significantly inhibited the secretion of IFNB, IL‐6, and TNF‐α induced by STING agonist DMXAA in mice (Figure S6A–F, Supporting Information). Notably, our results showed comparable amelioration in systemic inflammation by both compounds.

Next, we compared the potential cytotoxicity of CA and H‐151 in a variety of cells. Cell viability was assessed after adding different concentrations of CA or H‐151 (2.5‐40 µM) for 48 h to BMDMs, THP‐1, 293T, L929, and 3T3 cells, respectively. As shown in Figure S7A–E (Supporting Information), CA had no effect on cell viability after 48 h of drug administration, whereas H‐151 significantly inhibited cell viability, suggesting that CA has lower cytotoxicity than H‐151. Furthermore, our study showed that CA had high selectivity for STING signaling than H‐151. Because H‐151 inhibited the expression of p‐IRF3 triggered by STING‐independent stimulation of poly (I:C) as well as the downstream inflammatory response more potently than CA (Figure S7F–J, Supporting Information). Moreover, H‐151 also dramatically suppressed the mRNA levels of *Ifnb*, *Cxcl10*, and *Il‐6* induced by TLR ligand LPS compared to CA (Figure S7K–M, Supporting Information). Collectively, these data suggest that both CA and H‐151 exhibit comparable STING inhibitory activity, with CA has a better safety and specificity profile.

## 3 Discussion

*Rosmarinus officinalis L*. has displayed a wide range of medicinal and edible values, and numerous studies have evaluated its excellent role in antagonizing inflammation. However, the current understanding of its mechanism of action and biological activity remains largely unexplored, which limits its clinical application. Here, we demonstrated that CA, carnosol, and rosmanol, the main active ingredients from *Rosmarinus officinalis L*., dramatically block the innate immune cGAS‐STING‐mediated inflammatory response. Notably, further studies demonstrated that CA directly target the C‐terminal tail of STING, competitively inhibit the recruitment of TBK1 onto STING, and has remarkable anti‐inflammatory activity in vivo and in vitro, suggesting that CA can serve as a lead to design new therapeutics against STING‐driven diseases.

Given the critical role of the cGAS‐STING pathway in autoimmune and autoinflammatory diseases,^[^ ^4^ ^]^ targeting key proteins in this signaling is considered a promising therapeutic strategy in drug development. However, the medications targeting cGAS‐STING signaling are not available in clinics. Previous studies identified several STING antagonist, such as C176, C178,^[^ ^20^ ^]^ compound 18,^[^ ^37^ ^]^ myristic acid,^[^ ^38^ ^]^ and compound 13,^[^ ^39^ ^]^ but they are either inactive or have low bioactivity against human STING. In comparison, our study displayed a remarkable inhibitory effect of CA on human STING. Additionally, the currently reported STING antagonists can be broadly classified into the following categories: One is competitive inhibitors (such as SN‐011, Astin C)^[^ ^21^, ^40^ ^]^ that target the CDN binding site in ligand binding domain (LBD) of STING, the other is inhibitors that hinder STING oligomerization by covalently modifying the Cys^148^ residue of STING (such as licochalcone D and BB‐Cl‐amidine),^[^ ^22^, ^41^ ^]^ and another class of inhibitors that inhibit STING palmitoylation by covalently modifying Csy^88^/Csy^91^ of STING (such as Nitro‐fatty acids and H‐151).^[^ ^20^, ^42^ ^]^ By contrast, our study revealed a novel way in which drugs are bound to STING. Our results showed that CA directly bound with STING on its CTT, suggesting CA is a competitive antagonist in a way different from other reported antagonists. Moreover, the CTT of STING contains a IRF3 binding motif (362–366) and a TBK1 binding motif (369–377). ^[^ ^43^ ^]^ Our study displayed that CA lessened the interaction between TBK1 and STING but not affect the interaction of IRF3 and STING. As such, we speculate that CA may compete with TBK1 by targeting the TBK1 binding motif (369–377) on STING CTT. Future work about structural analysis will provide insights into the assumed mechanism.

For CDNs induced STING dimerization and trafficking, we guessed that CA might regulate STING trafficking and aggregation. The study showed that CA did not affect the oligomerization of STING induced by cGAMP, while CA dramatically inhibited either the aggregation of STING and TBK1 or the subcellular translocation of IRF3 and p65. A proposed model mentioned that second messenger cGAMP stimulates STING oligomerization, oligomerized STING recruits TBK1 to phosphorylate Ser366 of STING, leading to the classic activation of STING pathway ^[^ ^44^ ^]^. As indicated in Figure 3G, CA treatment decreased the phosphorylation of STING but did not interfere its oligomerization induced by cGAMP. As in Figure 4F,H, CA blocks the recruitment of TBK1 to STING signalosome. These findings suggest that CA might affect Ser366 phosphorylation of STING by competing for binding to STING with TBK1. The exact inhibition mechanism of STING via CA remains for further determination.

Notably, pull‐down assay showed that CA and its derivative carnosol precipitated only STING without binding cGAS, MAVS, TBK1 and IRF3 (Figure 4A–C), suggesting that CA and carnosol exert their inhibitory effects on this pathway by targeting STING. Moreover, this may explain why CA and carnosol specifically inhibited the activation of DNA‐sensing cGAS‐STING signaling, but had no effect on RNA‐sensing RIG‐MAVS pathway and TLR receptor pathway. Moreover, considering the potential clinical value of CA as a candidate for targeting STING, we further compared its inhibitory activity against STING with H‐151 in vivo. The results showed comparable amelioration in systemic inflammation mediated by aberrant activation of STING by both compounds. Additionally, CA might have better safety and specificity than H‐151, as H‐151 but not CA significantly reduced cell viability and induced cell death in vitro, while H‐151 showed some inhibitory effects on RIG‐I or TLR‐mediated signaling.

Previous SAR analyses suggested that the phenolic hydroxyl group contributes to the inhibitory activity of SN‐011 against STING.^[^ ^21^ ^]^ Given that CA has an abundant phenolic hydroxyl structure, we further evaluated the role of phenolic hydroxyl groups in the inhibition of STING activation by CA. The derivatives carnosol and rosmanol, which only substituted the carboxyl group on CA, also inhibited STING activation specifically, compared to CA. Conversely, when the phenolic hydroxyl groups on CA were substituted, its association with STING was abrogated and also its inhibitory activity on STING. This suggests that phenolic hydroxyl groups play a crucial role in CA inhibition of STING activation as well as downstream responses. Notably, previous studies have reported the role of polyphenols in anti‐inflammatory and antioxidant.^[^ ^45^, ^46^, ^47^ ^]^ In the future, it may be interesting to focus on the antagonistic effect of polyphenolic compounds on STING activity.

Overall, this study demonstrates that CA, a potent small molecule compound, specifically suppresses the cGAS‐STING pathway and innate inflammatory responses in vitro and in vivo. CA directly binds to STING CTT impairing the recruitment of TBK1 onto the STING signalosome and inhibiting the phosphorylation of STING and IRF3, then the nuclear translocation of IRF3 and p65. These results verify CA a promising lead compound for STING‐driven clinical disease and reveal that STING CTT may be a novel promising target for drug development.

## 4 Experimental Section

### Animals and Ethics Statement

*Sting^−/−^ and Trex1^−/−^* mice were obtained from The Jackson Laboratory (Shanghai, China). *Sting^−/−^* and *Trex1^−/−^* mice were bred by further mating the female and male *Sting^+/−^* and *Trex1^+/−^* mice, respectively. The mice were genotyped by standard PCR, using Quick Genotyping Assay Kit for Mouse Tail (D7283 M, Beyotime). Female WT mice (6‐8 weeks of age) were purchased from SPF Biotechnology Co., Ltd (Beijing, China). All animals were on C57BL/6 background and were raised under pathogen‐free conditions. The experimental protocol (Permit Number: IACUC‐2021‐0012) was approved by Animal Ethics Committee of the Fifth Medical Center, Chinese People's Liberation Army (PLA) General Hospital, Beijing, China.

### Antibodies and Reagents

Anti‐Phospho‐IRF3 (Ser396) (Cat#4947; lot#15) was purchased from Cell Signaling Technology (CST). Anti‐IRF3 (Cat#11312‐1‐AP; lot#001 31092), Anti‐STING (Cat# 19851‐1‐AP; lot#001 42695), Anti‐p65 (Cat#80979‐1‐RR), Anti‐Laminb1 (Cat#12987‐1‐AP; lot#10 004 949), Anti‐α‐Tubulin (Cat#66031‐1‐Ig; lot#10 028 345), Anti‐MAVS (Cat#66911‐1‐Ig; lot#00 076926), Anti‐β‐actin (Cat#60008‐1‐Ig; lot#10 041 142), Anti‐Flag (DDDDK tag) (Cat#66008‐4‐Ig; lot#10 041 988), and Anti‐GAPDH (Cat#60004‐1‐Ig; lot#10 029 187) were purchased from Proteintech. Anti‐HA‐tag pAb (Cat#561; lot#064) was obtained from MBL. Anti‐TBK1 (Cat#T55145; lot#T55145F) were purchased from Abmart. Anti‐HSP90 (Cat#F1132; lot#F113201) and Anti‐Phospho‐TBK1/NAK (Ser172) (Cat#F0255; lot#F025501) were from Selleck. Anti‐Strep‐Tag Il (Cat#HA500061; lot#HP0324) was obtained from HUABIO. Carnosic acid (Cat#T4957), Carnosol (Cat# T6S1302), and Rosmanol (Cat#T7033) were from TargetMol. HT‐DNA (Cat#D6898) and DMSO (Cat#D2650) were from Sigma‐Aldrich. DMXAA (Cat#HY‐10964), diABZI (Cat#HY‐112921B), and Mouse macrophage colony stimulating factor (M‐CSF) (Cat#HY‐P7085) were obtained from MedChemExpress (MCE). Phorbol‐12‐myristate‐13‐acetate (PMA) (Cat#tlrl‐pma), poly (I:C) (Cat#tlrl‐pic), poly (dA:dT) (Cat#tlrl‐patn), and lipopolysaccharide (LPS) (tlrl‐pglps) were from Invivogen. 2′3'‐cGAMP (cGAMP) (Cat#B8362) was from APEBIO. Strep‐Tactin^@^XT 4Flow^@^ (Cat#2‐5010‐010) was from IBA. Fetal bovine serum (FBS) (Cat#04‐001‐1ACS) and Opti‐MEM (Cat#31985‐670) were from Gibco. Interferon stimulatory DNA (ISD) was provided by Sangon Biotech (China). DNA oligonucleotides were shown in Table S1 (Supporting Information).

### Cell Lines

BMDMs were derived from WT, *Sting^−/−^*, or *Trex1^−/−^* mice. The procedure for obtaining and culturing BMDMs has been described previously.^[^ ^48^ ^]^ HEK293T, THP‐1, and L929 cells were kindly supported by Dr. Tao Li (National Center of Biomedical Analysis, NCBA). HEK293 cells (YC‐A008) and mouse embryonic fibroblasts (NIH/3T3, YC‐A013) were obtained from Ubigene (China). Cells have previously been tested for mycoplasma contamination. hPBMCs were isolated and cultured as described previously.^[^ ^49^, ^50^ ^]^ Briefly, the hPBMCs were isolated from peripheral blood of informed healthy volunteers using Ficoll‐Paque density gradient media (17 544 202, Cytiva). The experimental protocol (Permit Number: KY2023‐2‐10‐1) was approved by Animal Ethics Committee of the Fifth Medical Center, Chinese PLA General Hospital.

BMDMs were cultured in DMEM containing FBS (10%), penicillin/streptomycin (1%), and M‐CSF (25 ng mL^−1^) for 5 days. HEK293, HEK293T, 3T3, and L929 cells were cultured in DMEM containing FBS (10%) and penicillin/streptomycin (1%). hPBMCs and THP‐1 cells were cultured using 1640 medium with the same supplement. Unless otherwise indicated, BMDMs, THP‐1, hPBMCs, HEK293, HEK293T, 3T3, and L929 cells were seeded at densities of 1.0 × 10^6^ cells mL^−1^, 1.3 × 10^6^ cells mL^−1^, 1.3 × 10^6^ cells mL^−1^, 6 × 10^5^ cells mL^−1^, 6 × 10^5^ cells mL^−1^, 6 × 10^5^ cells mL^−1^, and 6 × 10^5^ cells mL^−1^, respectively. The cell densities were counted using TC10 Automated Cell Counter (BIO‐RAD).

### Plasmids and Transfection

Flag‐tagged cGAS, STING, IRF3, TBK1, and MAVS as well as HA‐tagged STING and IRF3 were provided by Dr. Tao Li (NCBA). Flag‐tagged cGAS, STING, IRF3, TBK1, and MAVS were constructed by inserting PCR‐amplified fragments into pcDNA3 vector (Invitrogen). The Flag‐tag was at the N‐terminus of STING, while at the C‐terminus of other proteins. HA‐tagged STING and IRF3 were constructed by inserting PCR‐amplified fragments into pCMV vector (YouBio). The HA‐tag was at the C‐terminus. Strep‐tagged STING and its mutants (1‐137, 138–379, 1–137+341‐379) were constructed using SnapGene and subsequently synthesized by YouBio (Hunan, China). Strep‐tagged STING and its mutants were constructed by inserting PCR‐amplified fragments into pCDH‐EF1‐MCS‐T2A‐Puro vector (YouBio). The Strep‐tag was at the N‐terminus. All plasmids were confirmed by sequencing and immunoblot. For transfection of plasmids into HEK293 and HEK293T cells, StarFect Transfection Reagent (Cat#C101, Genstar) was used, according to the manufacturer's instructions.

### Stimulants and Transfection

Stimulants in the experiments were used at the following concentrations: HT‐DNA, 2 µg mL^−1^; poly (I:C), 2 µg mL^−1^; poly (dA:dT), 2 µg mL^−1^; ISD, 2 µg mL^−1^; LPS, 10 µg mL^−1^; cGAMP, 2 µg mL^−1^; DMXAA, 15 µg mL^−1^; diABZI, 10 µg mL^−1^, CMA, 250 µg mL^−1^. ISD oligos were prepared as described previously.^[^ ^51^ ^]^ These stimulants were transfected into BMDMs, THP‐1, and hPBMCs with 3 µL of StarFect Transfection Reagent per 1 µg of HT‐DNA, poly (I:C), poly (dA:dT), ISD, and cGAMP. Cells were lysed and then harvested for immunoblotting assay after 2 h of HT‐DNA, poly (I:C), poly (dA:dT), ISD, cGAMP, DMXAA, diABZI, CMA stimulation, and 4 h of LPS stimulation. Cells were lysed and harvested for qPCR assay after 4 h of stimulation with these stimulants.

### Cell Viability Assay

BMDMs and THP‐1 cells were seeded into 96‐well plates, respectively. Next, CellTiter‐Glo Luminescent Cell Viability Assay (G7570, Promega) was used to evaluate the effect of different concentrations of CA on BMDMs and THP‐1 cells viability after 12 h of incubation. Cytotoxicity was quantified according to manufacturer's instructions. Similarly, to compare the safety of CA and H‐151, the viability of BMDMs, THP‐1, HEK293T, L929, and 3T3 cells was assessed by CellTiter‐Glo Luminescent Cell Viability Assay after treating these cells with vehicle control (DMSO, 1000x) or different concentrations of CA (0‐40 µM) and H‐151 (0‐40 µM) for 48 h.

### Immunoblot Assay

Total protein was extracted from cells with RIPA lysis buffer (P0013C, Beyotime) containing phosphatase inhibitor (C0002, TargetMol) and protease inhibitor (C0001, TargetMol), and then the protein quantity was determined using BCA Protein Assay Kit (P0012, Beyotime). The samples were prepared using SDS‐PAGE Sample Loading Buffer (P0015L, Beyotime). Subsequently, the samples were denatured at 105 °C for 15 min. For immunoblot assay, immunoprecipitates or whole‐cell lysates were loaded to SDS‐PAGE, typically with a load of 30 µL (30‐40 µg). Then, the proteins in the gel were transferred onto PVDF western blotting membranes and then blotted as described previously.^[^ ^52^, ^53^ ^]^

### Quantitative Real‐Time PCR

Quantitative real‐time PCR was performed as described previously.^[^ ^54^, ^55^ ^]^ Briefly, total RNA was extracted from cells and tissues by TRIzol Reagent (15596‐018, Invitrogen) according to the manufacturer's instructions. After cells and tissues were homogenized with TRIzol Reagent, 200 µL of chloroform was added and mixed by turning vigorously. The samples were allowed to stand at room temperature for 2 min before being centrifuged at 12 000 g, 4 °C for 15 min. The collected upper aqueous phase was precipitated with an equal volume of isopropanol at 4 °C for 10 min before centrifugation at 12 000 g, 4 °C for 10 min. The precipitated RNA microspheres were washed with 75% ethanol followed by centrifugation at 7500 g for 15 min, and after discarding the supernatant, it was air‐dried and dissolved in 30 µL of sterile, enzyme‐free water. Next, 1 µg of total RNA was reverse transcribed into cDNA using RT Master Mix for qPCR II (HY‐K0510A, MCE). Then, the qPCR was performed in10 µL reaction mixtures containing 25 ng cDNA, 5 µL SYBR Green qPCR Master Mix (2 x, HY‐K0522, MCE), and 5 µM primers. Relative quantification was carried out using the ∆∆CT method. Actin was served as an internal control. Specific primers (Tianyi Huiyuan, Beijing, China) used for quantitative real‐time PCR assays were shown in Table S2 (Supporting Information).

### STING Oligomerization Assay

The preparation of cell lysates and native gel electrophoresis for STING oligomerization assay has been described previously.^[^ ^40^, ^56^ ^]^ Briefly, BMDMs were pretreated with vehicle control (DMSO, 1000x) or CA for 1 h followed by stimulation with cGAMP for 2 h. Next, cells were lysed using cell lysate, after supernatant were harvested and native sample buffer (62.5 mM Tris‐Cl, 15% glycerol, and 1% deoxycholate, pH 6.8,) was added to prepare samples. Then, the samples were loaded to native‐PAGE gel, which was electrophoresed (25 mM Tris‐Cl, 192 mM glycine, pH 8.4, with or without 0.5% deoxycholate) at 80 mA for 1 h. Subsequently, the gel was soaked in SDS electrophoresis buffer for 30 min and the proteins in the gel were transferred to a PVDF membrane and analyzed by immunoblotting as previously described.

### Nuclear and Cytoplasmic Protein Extraction

BMDMs were pretreated with vehicle control (DMSO,1000x) or CA (10 µM) for 1 h and then stimulated with cGAMP or DMXAA for 2 h, respectively. After supernatants were completely removed, nuclear and cytoplasmic proteins were extracted using the Nuclear and Cytoplasmic Protein Extraction Kits (P0027, Beyotime), which was performed according to manufacturer's instructions. Nuclear translocation of IRF3 was assessed by western blot.

### Immunofluorescence

Immunofluorescence assay was carried out as described previously.^[^ ^52^ ^]^ For immunofluorescence detection of IRF3 or p65 nuclear translocation, THP‐1 cells or BMDMs were pretreated with vehicle control (DMSO,1000x) or CA (10 µM) for 1 h and then stimulated with diABZI for 2 h. Next, cells were fixed using 4% paraformaldehyde at 37 °C for 0.5 h and then permeabilized with PBS containing 0.25% Triton X‐100 at room temperature for 0.5 h. After blocked using rapid blocking solution (C200501, YangGuangBio), cells were stained with indicated primary antibodies as well as fluorescent‐conjugated secondary antibodies (P03S06S, Gene‐Protein Link). The cell nuclei were counterstained with Hoechst. Images were captured using a confocal microscope (TCS SP8 STED, Leica) at 60x magnification (oil‐immersion objective), following the manufacturer's instructions. The images were captured after previewing (512 × 512, 600 HZ) to find the most suitable focal plane for observation and the best dynamic range of image brightness. For quantification of IRF3 and p65 nuclear translocation, the total number of cells (more than 200 cells) and the number of positive cells from different visual fields of the same size were counted, and calculated the positive rate. The selection of statistical regions was random.

### Immunoprecipitation Assay

HEK293T cells in 6‐well plates were transfected with indicated plasmids for 18 h and then administrated with vehicle control (DMSO,1000x) or CA (20 µM) for 6 h. Then, the cells were lysed using 400 µL lysis buffer (50 mM Tris‐HCL, 1% Triton X‐100, 0.25% deoxycholate, 150 mM NaCl, 0.1% NP‐40, and 2 mM EDTA, pH7.4) containing 1% protease inhibitor cocktail. The harvested cell lysates were centrifuged for 15 minutes (12 000 g, 4 °C) and then immunoprecipitated with 4 µL Flag‐tagged affinity beads (A2220, Sigma‐Aldrich) or Strep‐tagged affinity beads (2‐5010‐010, IBA) for 4 h. The beads were washed four times using the corresponding lysis buffer and then resuspended with SDS‐loading buffer and boiled for 15 min. The precipitated proteins were evaluated by western blot.

### Synthesis of CA Derivatives Compound 1 (C1)

 

**Figure:**
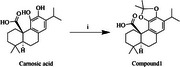
Synthesis of Compound 1: (i) TsOH, acetone, rt, 8 h.

P‐toluenesulfonic acid monohydrate (250 mg) and trimethoxymethane (3 mL) were added to a solution of CA (1 g, 9 mmol) in acetone (9 mL). The mixture was stirred for 8 h. Then the reaction was quenched by the addition of satd. aq. NaHCO_3_ to pH 7.0. The aqueous phase was extracted with ethyl acetate. The combined organic phase was dried over Na_2_SO_4_, filtered, and evaporated in vacuo. Chromatography on silica gel using petroleum ether: ethyl acetate (30: 1) as the eluent gave 1 (1.24 g, 90.2%) as a light yellow solid. Mp: 154.5–156.8 °C. ^1^H NMR (400 MHz, DMSO‐*d*_6_) δ 12.11 (s, 1H), 6.42 (s, 1H), 2.88 – 2.68 (m, 3H), 2.34 (dt, *J* = 12.1, 6.5 Hz, 1H), 2.03 (q, *J* = 13.5 Hz, 1H), 1.78 – 1.69 (m, 1H), 1.55 (d, *J* = 19.9 Hz, 6H), 1.44 (q, *J* = 10.9, 9.1 Hz, 3H), 1.30 – 1.21 (m, 2H), 1.15 (dd, *J* = 7.0, 3.3 Hz, 6H), 1.07 – 1.00 (m, 1H), 0.93 (s, 3H), 0.82 (s, 3H). ^13^C NMR (101 MHz, DMSO) δ 175.9, 144.6, 142.3, 130.4, 127.8, 119.3, 116.5, 52.9, 46.5, 40.6, 34.1, 32.7, 30.8, 28.4, 25.8, 25.7, 22.5, 22.4, 20.3, 20.2, 18.7.

### Coupled to Epoxy‐Activated‐Sepharose 6B

The coupling of CA and carnosol to Epoxy‐Activated‐Sepharose 6B (E6754, Sigma‐Aldrich) was performed according to the manufacturer's instructions. Briefly, 0.4 g of Epoxy‐Activated‐Sepharose 6B was wetted with deionized water and washed three times with coupling buffer (0.1 M Na_2_CO_3_, pH 9). Subsequently, Sepharose 6B was incubated with CA or carnosol at a concentration of 10 mg mL^−1^ (400 uL DMSO + 800 uL coupling buffer) overnight at 37 °C with rotation. After repeatedly washing away the free CA or carnosol, the CA‐ or carnosol‐conjugated Sepharose 6B was obtained.

### Coupled to EAH Sepharose 4B

The coupling of CA and C1 to EAH Sepharose 4B (17 056 901, Cytiva) was performed according to the manufacturer's instructions. Briefly, the required amount of Sepharose 4B was supplied preswollen in 20% ethanol and washed three times with distilled water (pH 5.0). Subsequently, Sepharose 4B was incubated with 10 mg mL^−1^ of CA or C1 by carbodiimide coupling method at room temperature by rotary incubation overnight (The final concentration of carbodiimide was 0.1 M, pH 5.0).

### CA Pull‐Down Assay

The indicated densities of BMDMs or HEK293T cells (transfected with indicated plasmids) were lysed using the cell lysis buffer (50 mM Tris‐HCL, 1% Triton X‐100, 0.25% deoxycholate, 150 mM NaCl, 0.1% NP‐40, and 2 mM EDTA, PH7.4). Then, the harvested cell lysates were centrifuged at 12 000 g and 4 °C for 15 min. After that, the cell lysates were incubated with CA (or carnosol)‐conjugated epoxy activated Sepharose 6B or CA (or C1)‐conjugated EAH Sepharose 4B at 4 °C for 4 h. Next, the beads were washed using the corresponding lysis buffer for ten times. The CA‐conjugated proteins were separated using SDS‐loading buffer and detected by immunoblot assay. The harvested cell lysates needed to be briefly ultrasound (ice bath conditions, ultrasound 3 s with 6 s interval, repeated 3 times) using an ultrasonic cell crusher (VCX130, SONICS, AMPL 20%) before incubation with CA‐conjugated Sepharose 6B. Additionally, the cell lysates were incubated with control epoxy activated Sepharose 6B for 6 hours before incubating with CA‐conjugated epoxy activated Sepharose 6B to avoid possible interference.

### Surface Plasmon Resonance (SPR) Assay

For SPR assay, the binding affinity of CA or carnosol to purified human STING protein (TP308418, protein no: Q86WV6, OriGene) was determined using a Biacore T200 SPR instrument (GE, Health care) at 25 °C. All SPR‐based materials were purchased from GE Healthcare. Protein was diluted in HEPES buffered saline‐EP (HBS‐EP; GE Healthcare) and immobilized on an SA chip. Approximately 17 852 resonance units (RU) of the immobilized peptides were obtained. Interaction analyses were tested using HBS‐EP as a running buffer. Increasing concentrations of PHF20L1 TUDOR (0.3125, 0.625, 1.25, 2.5, and 5 uΜ) were injected using the “Kinetics/Affinity” program. A flow cell without immobilized peptide served as a nonspecific binding control. The SA chip surface was regenerated after each cycle by injecting 10 mM NaOH for 30 s. *Ka*, *Kd*, and *KD* were determined using the “Kinetics” model in the Biacore T200 evaluation software version 2.0.

### Microscale Thermophoresis (MST) Assay

The MST assay was performed as described previously.^[^ ^40^ ^]^ Briefly, purified human STING protein was labeled using the Monolith His‐Tag Labeling Kit (MO‐L018, NanoTemper Technologies). Subsequently, CA and C1 were diluted to different concentrations by buffer and mixed with labeled STING and incubated for 30 min at room temperature. After that, samples were loaded into standard glass capillaries and analyzed by thermophoresis on a NanoTemper Monolith NT.115 instrument (NanoTemper Technologies). The MST curves were fitted by MO Affinity Analysis v2.3 software.

### Enzyme Linked Immunosorbent Assay (ELISA)

The levels of TNF‐α, IL‐6, and IFN‐β in mouse serum or peritoneal lavage fluid (PLF) were measured using Mouse TNF‐α ELISA Kit (1 217 202, Dakewe), Mouse IL‐6 ELISA Kit (1 210 602, Dakewe), and IFNβ bioluminescent ELISA kits (luex‐mifnbv2, Invivogen), respectively. Follow the manufacturer's instructions.

### In Vivo Experiments

WT C57BL/6 mice (female, 6–8 weeks old) were injected intraperitoneally with CA (15 mg kg^−1^ and 30 mg kg^−1^) or its vehicle (10% DMSO, 10% Tween 80 and 80% saline, 200 uL) for 2 h followed by treatment with DMXAA (30 mg kg^−1^) or its vehicle (10% DMSO, 40% PEG300, and 50% saline, 200 uL) for 5 h (intraperitoneal injection). Then, the mouse serum and PLF were collected to assess the levels of inflammatory factors such as IFNB, TNF‐α, and IL‐6. Furthermore, mRNA levels of *Ifnb*, *Il‐6*, *Cxcl10*, and *Tnf‐α* in PLF were also detected.

In the second experiment, four‐week‐old *Trex1^−/−^* or WT mice were injected intraperitoneally with CA (20 mg kg^−1^) or its vehicle for 14 consecutive days. Next, tissues (heart, tongue, muscle, stomach, and kidney) were collected for mRNA expression determination and H&E staining.

To compare the therapeutic effects of CA and H‐151 in vivo, 6–8 weeks old female C57BL/6 mice were pretreated with 20 mg kg^−1^ of CA and H‐151 intraperitoneally for 2 h, respectively, and then treated with 30 mg kg^−1^ DMXAA for 5 h. Subsequently, mouse serum and PLF were harvested and levels of inflammatory factors IFN‐β, TNF‐α, and IL‐6 were assessed by corresponding ELISA kits.

### Statistical Analysis

Statistical analysis was executed by Excel and GraphPad Prism 6 software. One‐way ANOVAs, followed by Dunnett's post hoc test, was used to perform the comparisons among multiple groups. An unpaired two‐tailed Student's *t* test was used for statistical analysis of two independent treatments. Data were displayed as mean ± s.d. unless indicated otherwise*. P*‐values < 0.05 were considered statistically significant.

## Conflict of Interest

The authors declare no conflict of interest.

## Author Contributions

W.M., G.X., L.L., and J.W. contributed equally to this work. W.M.: conceptualization; investigation; methodology; visualization; data curation and analysis; validation; original manuscript writing and review. G.X., and L.L.: data analysis; supervision; investigation; original manuscript writing and review. J.W.: data curation and analysis; validation; investigation. J.Z., Y.X., and Z.W.: methodology; visualization; supervision. T.L., W.L., and H.Y.: investigation; visualization. Z.W., and X.Z.: methodology; supervision. G.X., X.X., and Z.B.: conceptualization; project administration; resources; supervision; original manuscript review. All authors read and approved the final version of the manuscript.

## Supporting information



Supporting Information

## Data Availability

The data that support the findings of this study are available from the corresponding author upon reasonable request.
