# Supplementary material for: Carnosic Acid Directly Targets STING C‐Terminal Tail to Improve STING‐Mediated Inflammatory Diseases
Source: Adv Sci (Weinh). 2025 Feb 18;12(14):2417686. doi: 10.1002/advs.202417686 (PMC11984877; doi:10.1002/advs.202417686)

**Supplementary information**

**Carnosic Acid Directly Targets STING C-terminal Tail to Improve STING-Mediated Inflammatory Diseases**

*Wenqing Mu, Guang Xu^*^, Ling Li, Jincai Wen, Ye Xiu, Jia Zhao, Tingting Liu, Ziying Wei, Wei Luo, Huijie Yang, Zhixin Wu, Xiaoyan Zhan, Xiaohe Xiao^*^, Zhaofang Bai^*^*

**Figure S1**

**
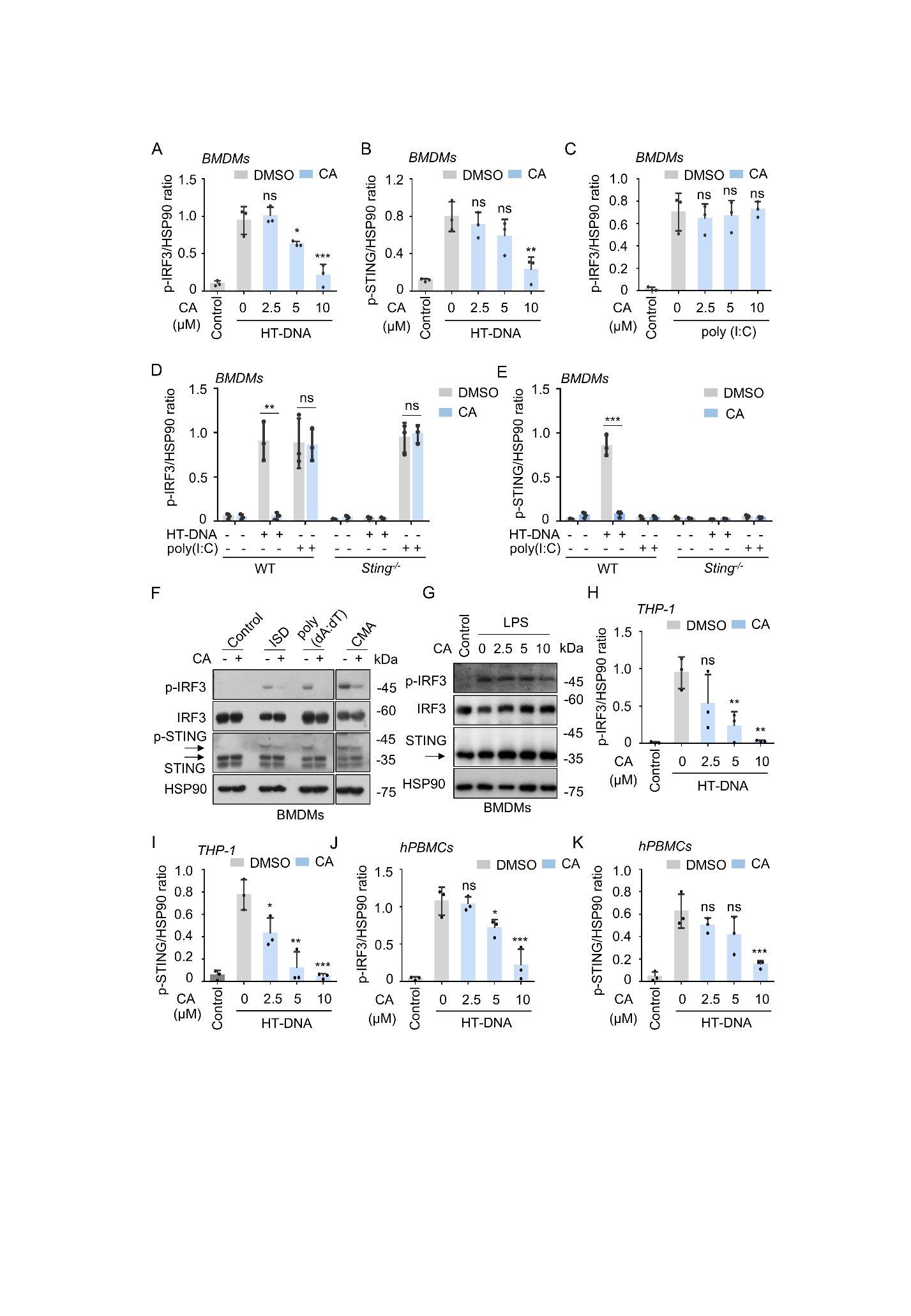
**

**Figure S1. CA Selectively Blocks the Cytoplasmic DNA-Triggered cGAS-STING Signaling Activation and Downstream Gene Expression.** A-C) BMDMs were administrated with vehicle control (DMSO) or the indicated concentrations of CA for 1 hour followed by stimulation with HT-DNA (2 μg ml^-1^) or poly (I:C) (2 μg ml^-1^) for 2 hours, respectively. Band intensities of p-IRF3 (A, C) and p-STING (B) were quantified by gray scale analysis. D, E) WT BMDMs and *Sting^-/-^* BMDMs were pretreated with vehicle control (DMSO) or CA (10 μM) for 1 h follow by stimulation with HT-DNA (2 μg ml^-1^) or poly (I:C) (2 μg ml^-1^) for 2 h, respectively. Band intensities of p-IRF3 (D) and p-STING (E) were quantified by gray scale analysis. F) BMDMs were treated with ISD (2 μg ml^-1^), poly (dA:dT) (2 μg ml^-1^), and CMA (250 μg ml^-1^) for 2 h in the presence or absence of CA (10 μM). The p-IRF3 and p-STING were evaluated by western blot. G) BMDMs were administrated with CA (2.5, 5, 10 μM) for 1 hour followed by stimulation with LPS (10 μg ml^-1^) for 4 hours. The p-IRF3 and p-STING were evaluated by western blot. H,I) THP-1 cells were treated with CA (2.5, 5, 10 μM) for 1 h followed by stimulation with HT-DNA (2 μg ml^-1^) for 2 h. Band intensities of p-IRF3 (H) and p-STING (I) were quantified by gray scale analysis. J,K) hPBMCs were treated with CA (2.5, 5, 10 μM) for 1 h followed by stimulation with HT-DNA (2 μg ml^-1^) for 2 h. Band intensities of p-IRF3 (J) and p-STING (K) were quantified by gray scale analysis. HSP90 was served as a loading control. Data were expressed as the mean ± s.d. (n=3); **P <* 0.05, **P <* 0.01, ****P* *<* 0.001; ns, no significance. (A-C) and (H-K) One-Way ANOVA followed by the Dunnett's post hoc test. (D,E) Statistics differences were analyzed using an unpaired Student’s *t*-test.

**Figure S2**


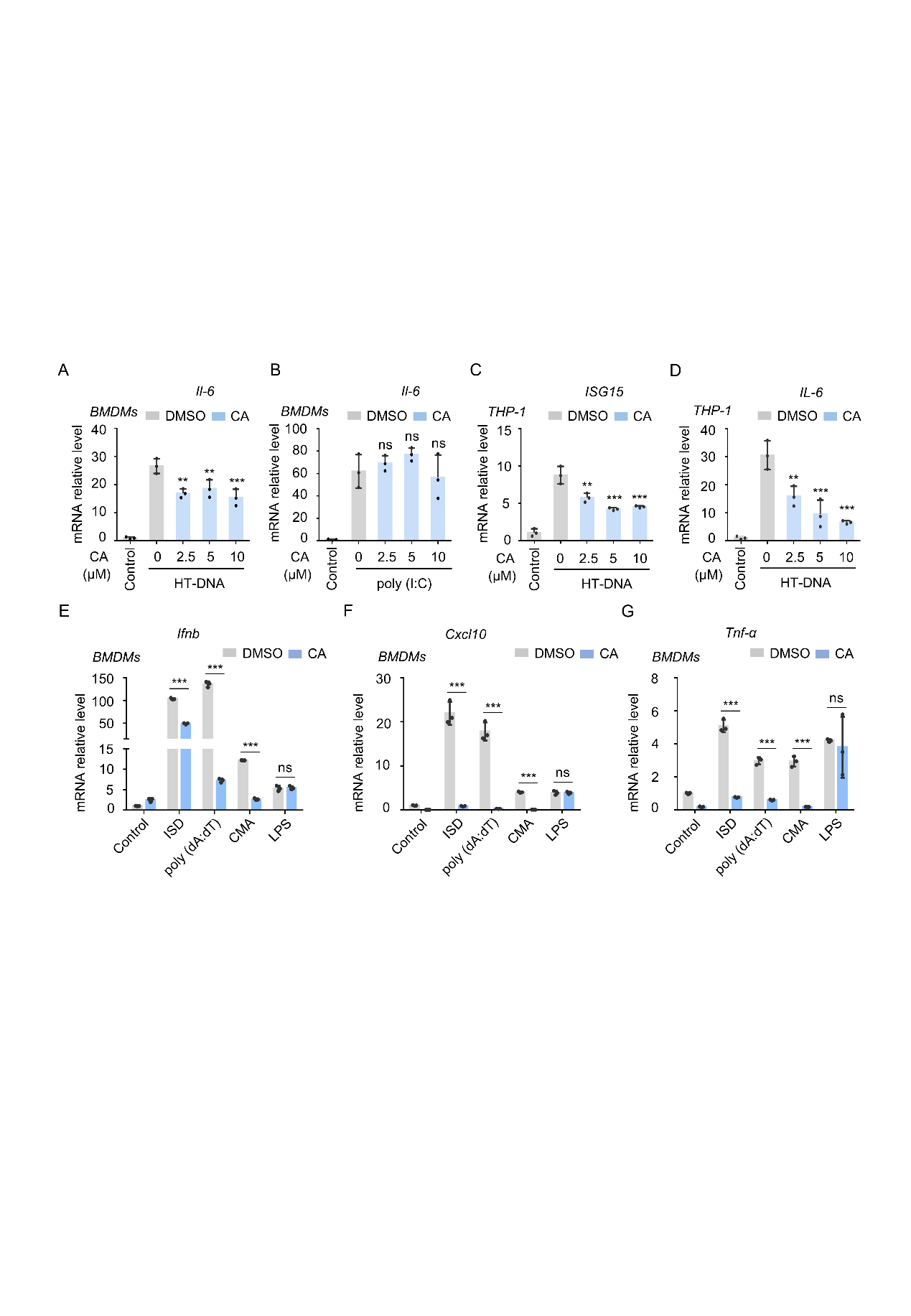


**Figure S2. CA Selectively Blocks Cytoplasmic DNA-Triggered Expression of Interferon-related and Inflammatory Genes.** A, B) qPCR analysis of *Il-6* mRNA levels from BMDMs pretreated with DMSO or CA (2.5, 5, 10 μM) for 1 hour followed by stimulation with HT-DNA (2 μg ml^-1^) (A) or poly (I:C) (2 μg ml^-1^) (B) for 4 hours. C, D) qPCR analysis of *ISG15* (C) and *IL-6* (D) mRNA levels from THP-1 cells pretreated with DMSO or CA (2.5, 5, 10 μM) for 1 hour followed by stimulation with HT-DNA for 4 hours. E-G) BMDMs were administrated with CA (10 μM) for 1 hour followed by stimulation with ISD (2 μg ml^-1^), poly(dA:dT) (2 μg ml^-1^), CMA (250 μg ml^-1^), and LPS (10 μg ml^-1^) for 4 hours. The mRNA levels of *Ifnb* (E)*, Cxcl10* (F)*,* and *Tnf-α* (G) were measured by qPCR assay. Data are presented as mean ± s.d. (n=3). ***P* < 0.01, ****P* < 0.001; ns, no significance. Gene expression was normalized to β-Actin. (A-D) One-Way ANOVA followed by the Dunnett's post hoc test. (E-G) Statistics differences were analyzed using an unpaired Student’s *t*-test.

**Figure S3**


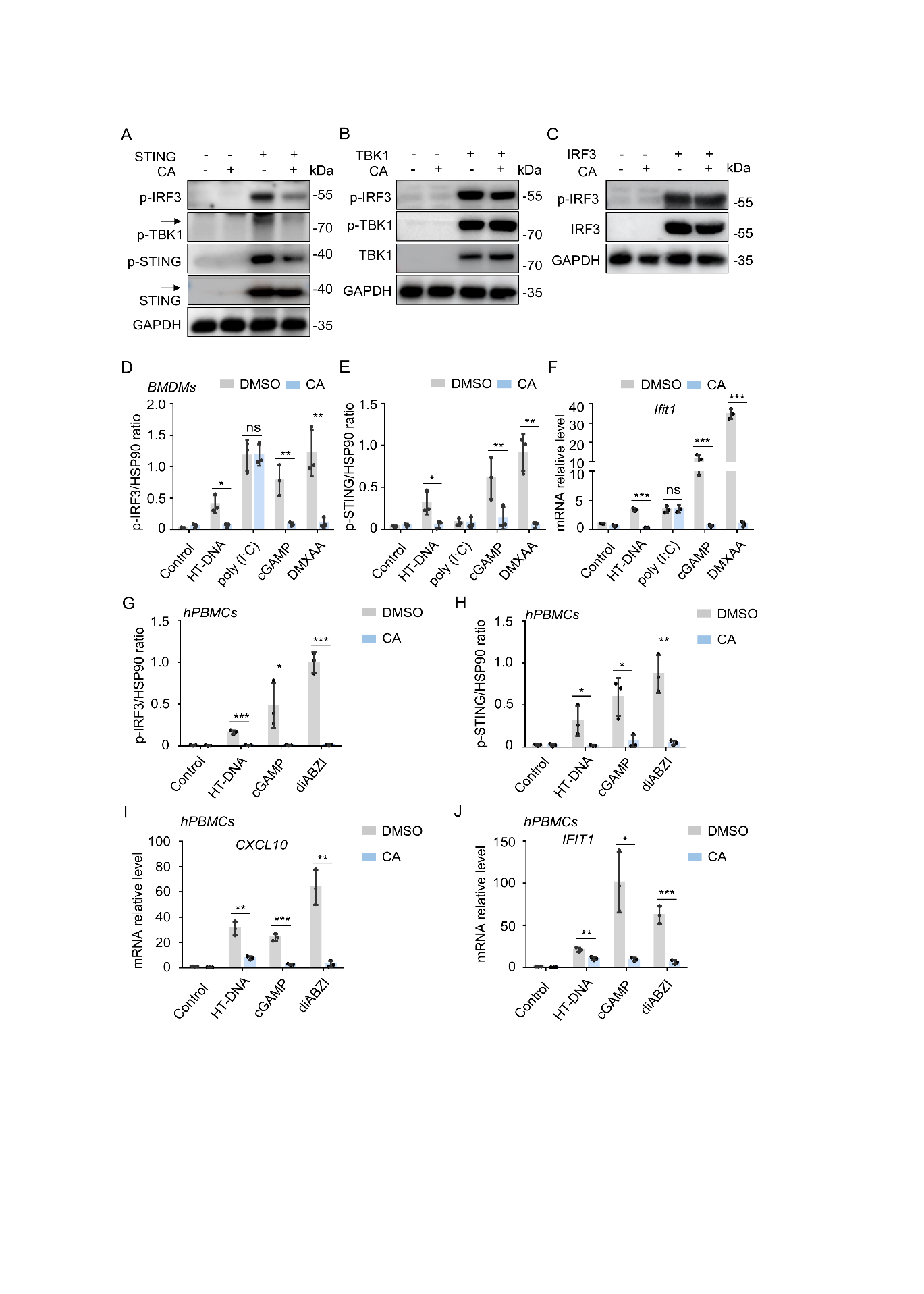


**Figure S3. CA Directly Regulates STING Signalosome Activation.** A) Immunoblot analysis of p-IRF3, p-TBK1, and p-STING from HEK293 cells transfected with Strep-tagged STNG for 6 h, followed by administration with CA (20 μM) for 18 h. B) Immunoblot analysis of p-IRF3 and p-TBK1 from HEK293 cells transfected with Flag-tagged TBK1 for 6 h, followed by administration with CA (20 μM) for 18 h. C) Immunoblot analysis of p-IRF3 from HEK293 cells transfected with Flag-tagged IRF3 for 6 h, followed by administration with CA (20 μM) for 18 h. D, E) BMDMs were pretreated with vehicle control (DMSO) or CA (10 μM) for 1 hour followed by stimulation with HT-DNA (2 μg ml^-1^), poly (I:C) (2 μg ml^-1^), cGAMP (2 μg ml^-1^), or DMXAA (15 μg ml^-1^) for 2 hours. Band intensities of p-IRF3 (D) and p-STING (E) were quantified by gray scale analysis. F) qPCR analysis of *Ifit1* (F) from BMDMs pretreated with CA (10 μM) for 1 hour followed by treatment with the indicated stimulants for 4 hours. G, H) hPBMCs were treated with CA (10 μM) for 1 h followed by stimulation with HT-DNA (2 μg ml^-1^), cGAMP (2 μg ml^-1^) and diABZI (10 μg ml^-1^) for 2 h. Band intensities of p-IRF3 (G) and p-STING (H) were quantified by gray scale analysis. I, J) qPCR analysis of *CXCL10* (I) and *IFIT1* (J) from hPBMCs pretreated with CA (10 μM) for 1 hour followed by treatment with the indicated stimulants for 4 hours. (D, E, G, H) HSP90 was served as a loading control. (F, I, J) Gene expression was normalized to β-Actin. Data are presented as mean ± s.d. (n=3). **P* < 0.05, ***P* < 0.01, ****P* < 0.001; ns, no significance. Statistics differences were analyzed using an unpaired Student’s *t*-test.

**Figure S4**


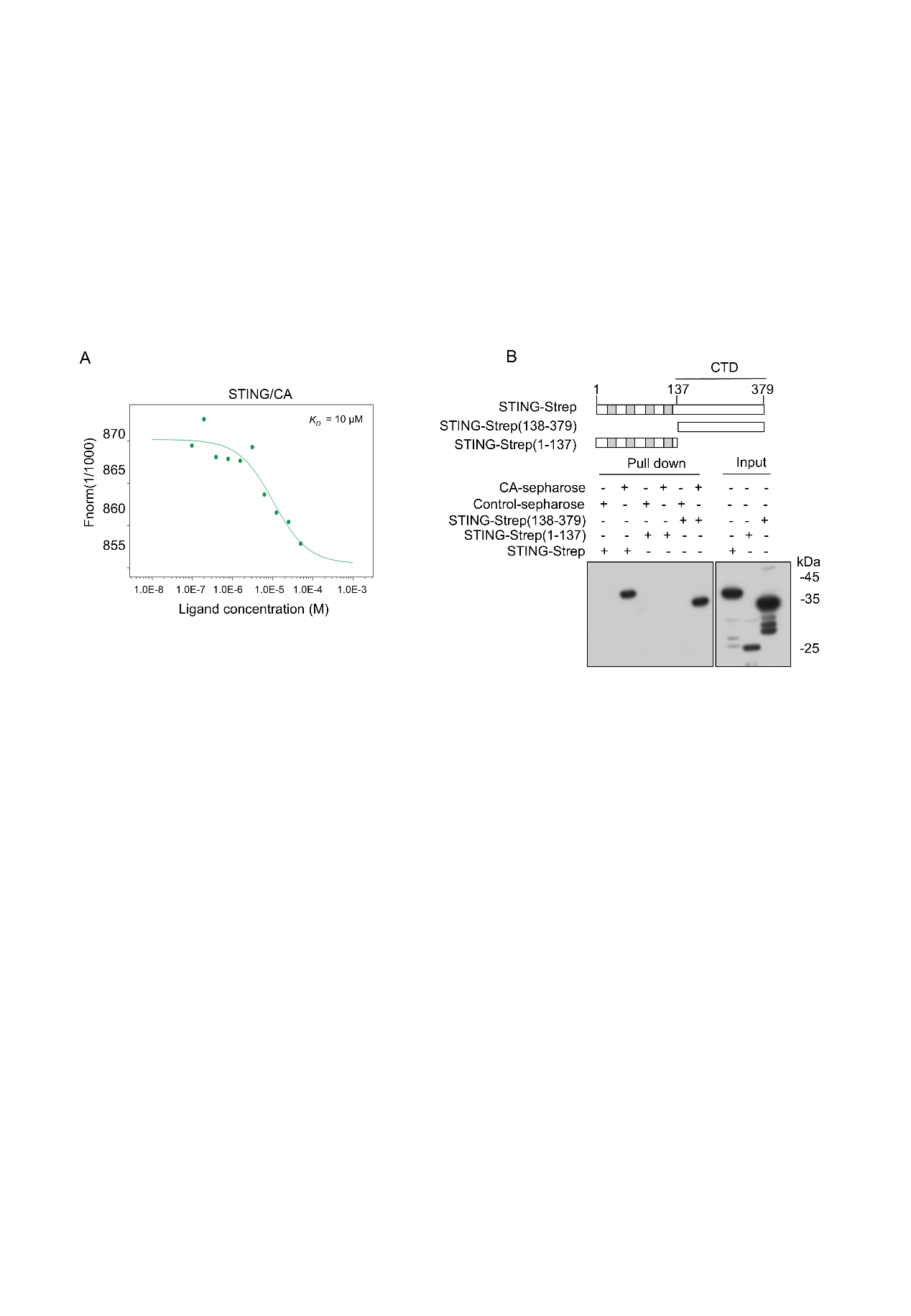


**Figure S4. CA Directly Binds to STING.** A) The kinetics of CA binding to STING were assessed by microscale thermophoresis (MST) assay. B) Schematic of STING and its truncation mutants (top). HEK293T cells were transfected with strep-tagged STING and its mutants (1-137 or 138-379) for 24 hours, respectively. Then cell lysates were harvested and incubated with control-sepharose or CA-sepharose. The pull-down samples were analyzed using immunoblot.

**Figure S5**


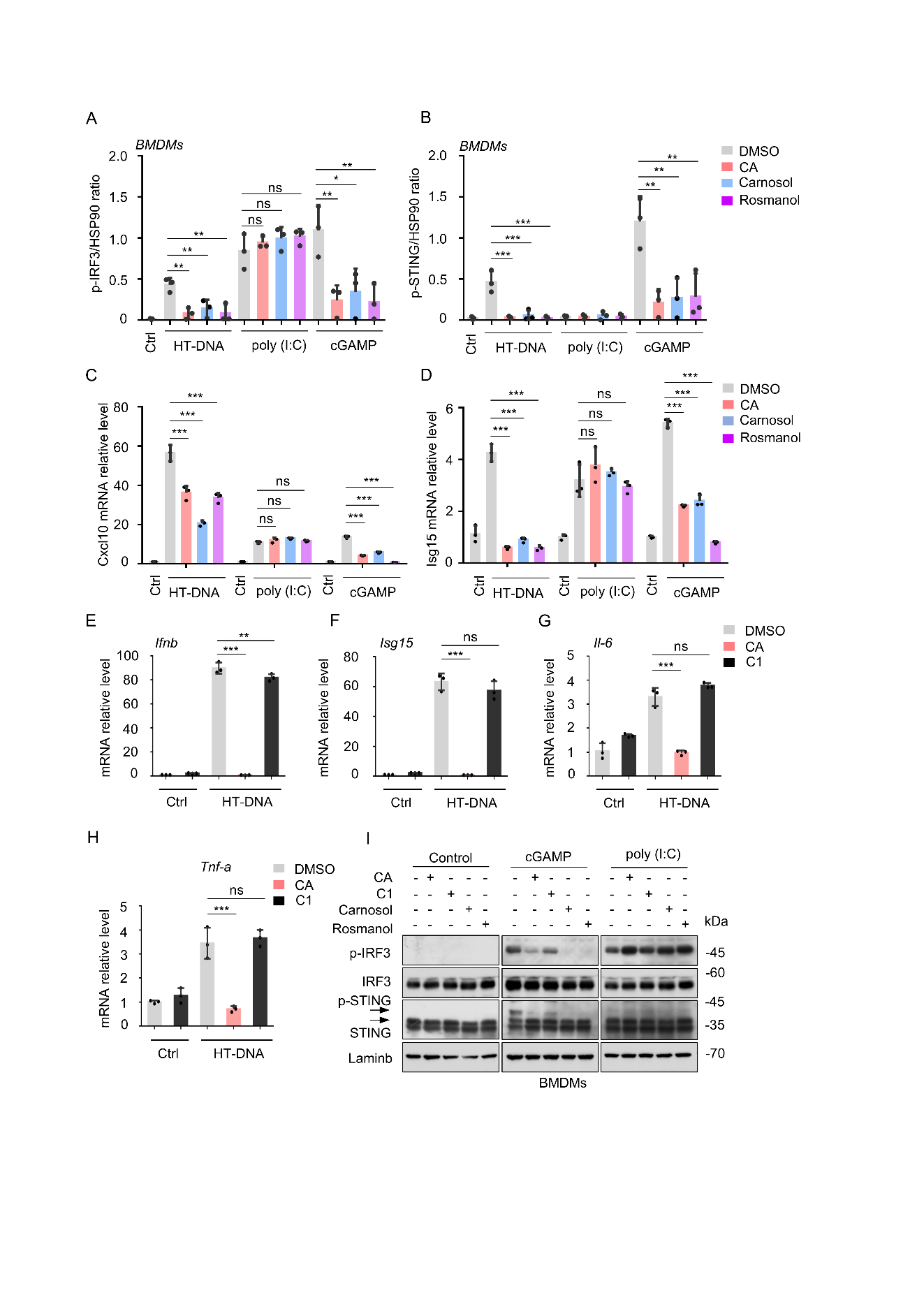


**Figure S5. The Phenolic Hydroxyl Groups Are Essential for CA-Mediated STING Inhibitory Activity.** A, B) BMDMs were pretreated with vehicle control (DMSO) or CA (10 μM), carnosol (10 μM), and rosmanol (10 μM) for 1 hour followed by stimulation with HT-DNA (2 μg ml^-1^), poly (I:C) (2 μg ml^-1^), or cGAMP (2 μg ml^-1^) for 2 hours. Band intensities of p-IRF3 (A) and p-STING (B) were quantified by gray scale analysis. C, D) qPCR analysis of *Cxcl10* (C) and *Isg15* (D) from BMDMs administrated with CA (10 μM), carnosol (10 μM), or rosmanol (10 μM) for 1 hour followed by stimulation with HT-DNA (2 μg ml^-1^), poly (I:C) (2 μg ml^-1^), or cGAMP (2 μg ml^-1^) for 4 hours. E-H) BMDMs were treated with CA (10 μM) and C1 (10 μM) for 1 h followed by stimulation with HT-DNA (2 μg ml^-1^) for 4 h. The mRNA levels of *Ifnb* (E), *Isg15* (F), *Il-6* (G), and *Tnf-a* (H) were evaluated by qPCR assay. I) BMDMs were treated with CA (10 μM) and its derivatives carnosol (10 μM), rosmanol (10 μM), and C1 (10 μM) for 1 h followed by stimulation with cGAMP (2 μg ml^-1^) or poly (I:C) (2 μg ml^-1^) for 2 h. The expression of p-IRF3 and p-STING were evaluated by western blot. Data are presented as mean ± s.d. (n=3). (A, B) HSP90 was served as a loading control. (C-H) Gene expression was normalized to β-Actin. **P* < 0.05, ***P* < 0.01, ****P* < 0.001; ns, no significance. One-Way ANOVA followed by the Dunnett's post hoc test.

**Figure S6**


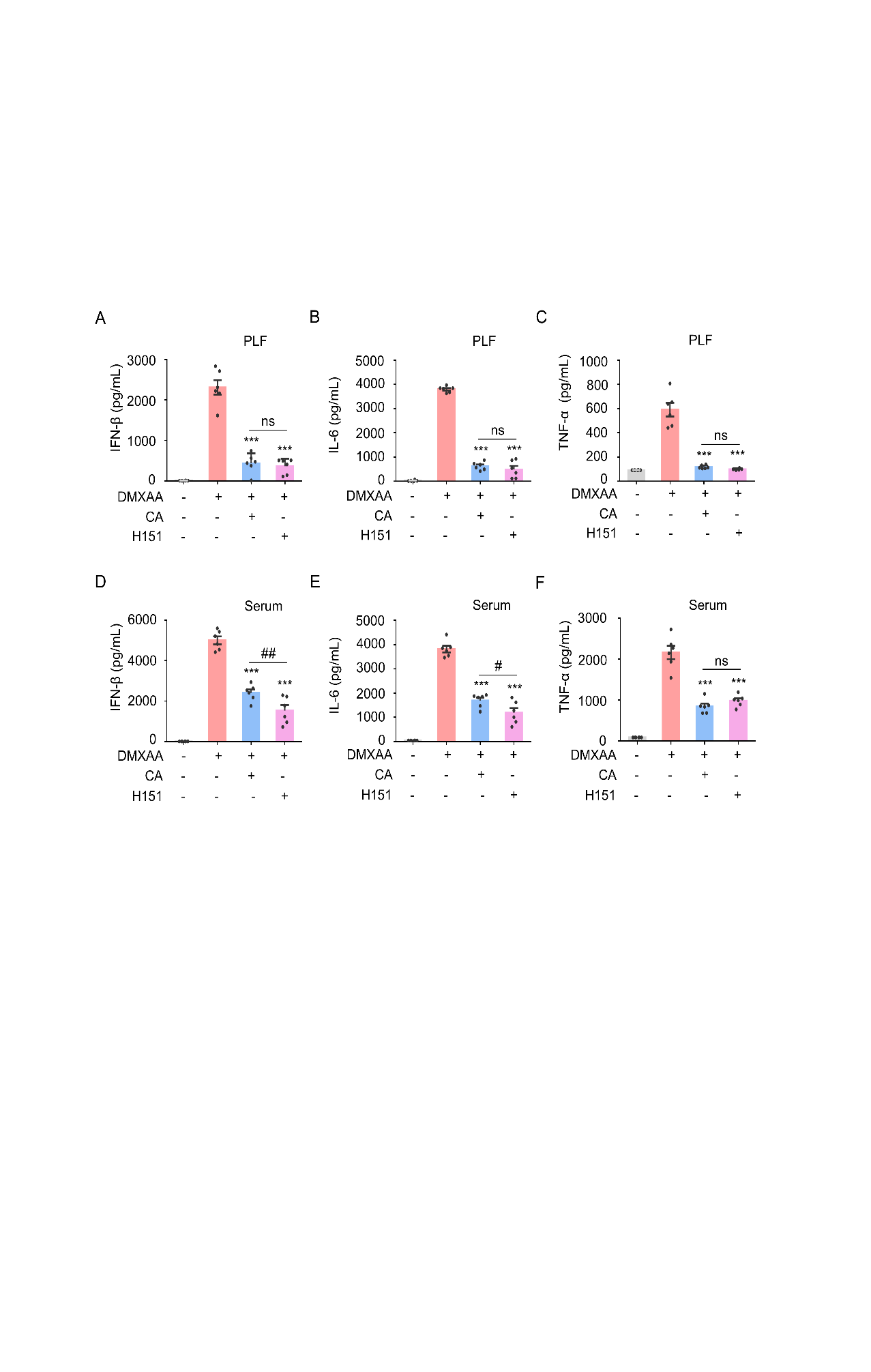


**Figure S6. Comparable Amelioration in STING-Induced Systemic Inflammation by CA and H-151.** A-F) Female C57/BL6 mice were administrated with the control vehicle (10% DMSO, 10% Tween 80 and 80% saline) or CA (20 mg kg^-1^) and H-151 (20 mg kg^-1^) for 2 hours followed by treatment with the control vehicle (10% DMSO, 40% PEG300, and 50% saline, 200 ul) or DMXAA (30 mg kg^-1^) for 5 hours. ELSA analysis of IFN-β (A), IL-6 (B), and TNF-α (C) in mouse peritoneal lavage fluid (PLF) as well as IFN-β (D), IL-6 (E), and TNF-α (F) in mouse serum. Data from (A-F) are presented as mean ± s.e.m. (n=6). ****P* < 0.001 *vs*. DMXAA group; *^#^P* < 0.05, *^##^P* < 0.01 *vs*. DMXAA + H-151 group; ns, no significance. One-Way ANOVA followed by the Dunnett's post hoc test.

**Figure S7**


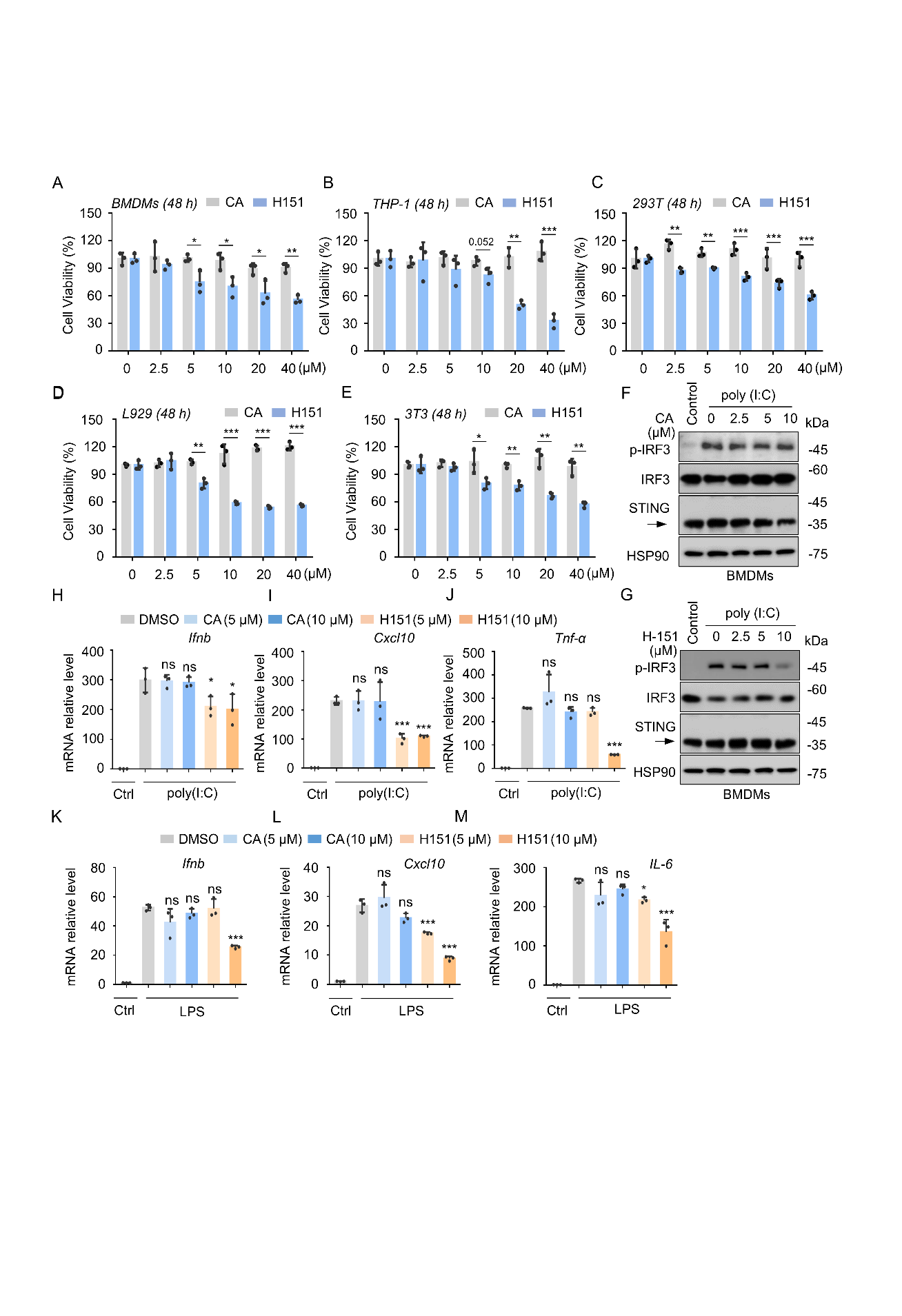


**Figure S7. CA Exhibits Low Cytotoxicity and High Specificity in Comparison with H-151.** A-E) BMDMs (A), THP-1 (B), 293T (C), L929 (D), and 3T3 cells (E) were incubated with the indicated concentration of CA or H-151 (2.5-40 μM) for the indicated time periods. Cell viability was measured by MTS assay. F, G) BMDMs were treated with the indicated concentration of CA (F) or H-151 (G) for 2 h followed by stimulation with poly (I:C) (2 μg ml^-1^) for 2 h. The expression of p-IRF3 were assessed by western blot. H-J) BMDMs were treated with the indicated concentration of CA or H-151 for 2 h followed by stimulation with poly (I:C) (2 μg ml^-1^) for 4 h. The mRNA levels of *Ifnb* (H), *Cxcl10* (I), and *Tnf-α* (J) were assessed by qPCR assay. (K-M) BMDMs were treated with the indicated concentration of CA or H-151 for 2 h followed by stimulation with LPS (10 μg ml^-1^) for 4 h. The mRNA levels of *Ifnb* (K), *Cxcl10* (L), and *Il-6* (M) were assessed by qPCR assay. Data from (A-E) are presented as mean ± s.d. (n=3). **P* < 0.05, ***P* < 0.01, ****P* < 0.001. *P* values were calculated by unpaired two-tailed Student’s *t* test. Data from (H-M) are presented as mean ± s.d. (n=3). **P* < 0.05, ***P* < 0.01, ****P* < 0.001, ns, not significant. *P* values were calculated by One-way ANOVAs, followed by Dunnett's post hoc test.

**Table S1.** **DNA Oligonucleotides Sequences**

| **Name** | **Prime** | **Sequence (5′-3′)** |
| --- | --- | --- |
| ISD | Forward | TACAGATCTACTAGTGATCTATGACTGATCTGTACATGATCTACA |
|  | Reverse | TGTAGATCATGTACAGATCAGTCATAGATCACTAGTAGATCTGTA |

**Table S2.** **Sequences of PCR Primers Used in this Study**

| **Name** | **Prime** | **Sequence (5′-3′)** |
| --- | --- | --- |
| mActin | Forward | GGCTGTATTCCCCTCCATCG |
|  | Reverse | CCAGTTGGTAACAATGCCATGT |
|  |  |  |
| mIFNB | Forward | TCCGAGCAGAGATCTTCAGGAA |
|  | Reverse | TGCAACCACCACTCATTCTGAG |
|  |  |  |
| mCXCL10 | Forward | ATCATCCCTGCGAGCCTATCCT |
|  | Reverse | GACCTTTTTTGGCTAAACGCTTTC |
|  |  |  |
| mIL-6- | Forward | CACTTCACAAGTCGGAGGCT |
|  | Reverse | CTGCAAGTGCATCATCGTTGT |
|  |  |  |
| mISG15 | Forward | GGTGTCCGTGACTAACTCCAT |
|  | Reverse | CTGTACCACTAGCATCACTGTG |
|  |  |  |
| mIFIT1 | Forward | GAACCCATTGGGGATGCACAACCT |
|  | Reverse | CTTGTCCAGGTAGATCTGGGCTTCT |
|  |  |  |
| mTNFα | Forward | GGGCAGTTAGGCATGGGAT |
|  | Reverse | TGAGCCTTTTAGGCTTCCCAG |
|  |  |  |
| hActin- | Forward | CATGTACGTTGCTATCCAGGC |
|  | Reverse | CTCCTTAATGTCACGCACGAT |
|  |  |  |
| hIFNB | Forward | TCCAAATTGCTCTCCTGTTG |
|  | Reverse | GCAGTATTCAAGCCTCCCAT |
|  |  |  |
| hCXCL10 | Forward | TGGCATTCAAGGAGTACCTC |
|  | Reverse | TTGTAGCAATGATCTCAACACG |
|  |  |  |
| hIL-6- | Forward | ACTCACCTCTTCAGAACGAATTG |
|  | Reverse | CCATCTTTGGAAGGTTCAGGTTG |
|  |  |  |
| hISG15 | Forward | CGCAGATCACCCAGAAGATCG |
|  | Reverse | TTCGTCGCATTTGTCCACCA |
|  |  |  |
| hIFIT1- | Forward | GCGCTGGGTATGCGATCTC |
|  | Reverse | CAGCCTGCCTTAGGGGAAG |
|  |  |  |
| hTNFα | Forward | CCTCTCTCTAATCAGCCCTCTG |
|  | Reverse | GAGGACCTGGGAGTAGATGAG |

**Uncropped Blots for Data in Figure 1-5, Figure S1,S3-S5, and S7**

**Uncropped Blots for Figure 1**


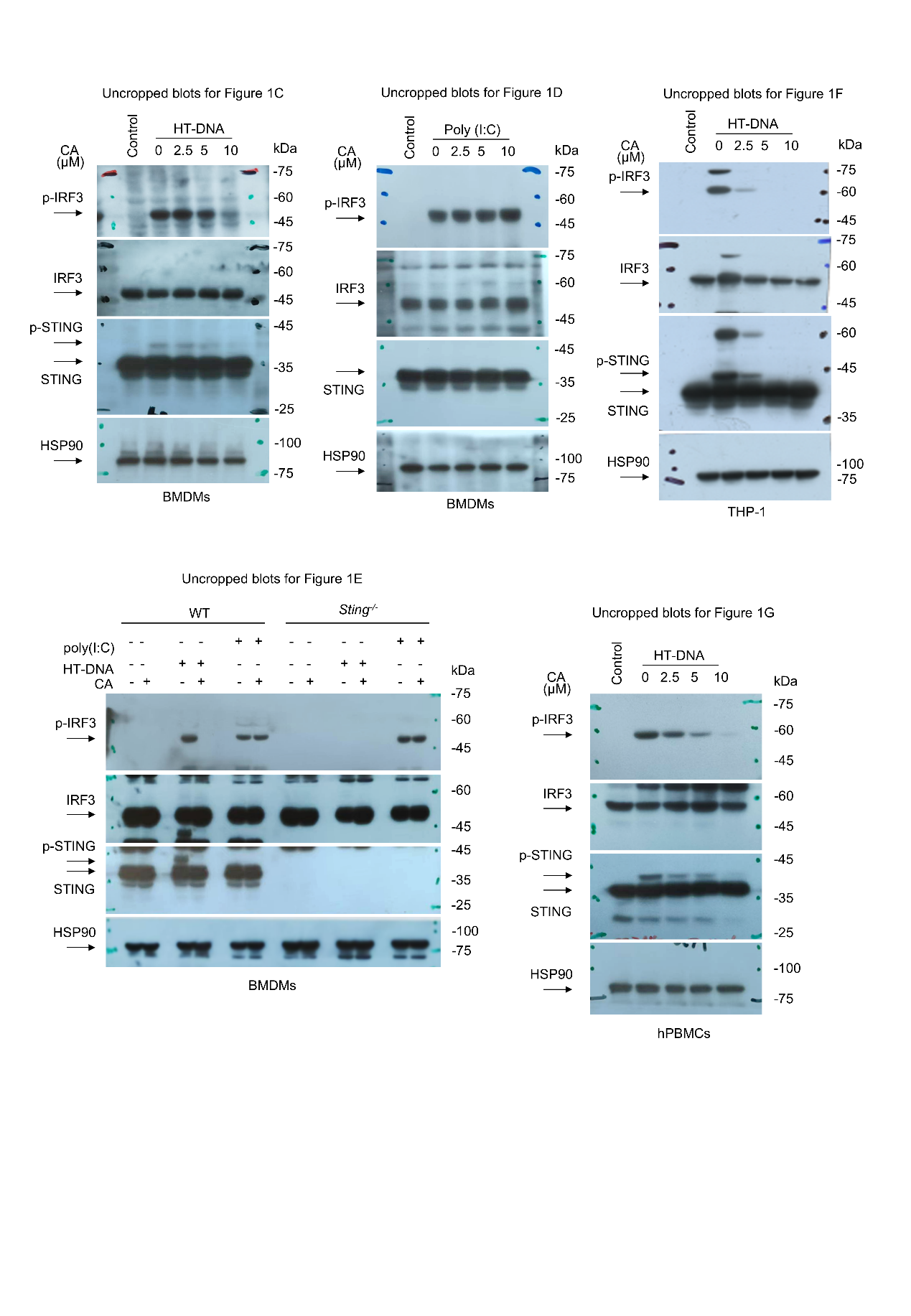


**Uncropped Blots for Figure 2**


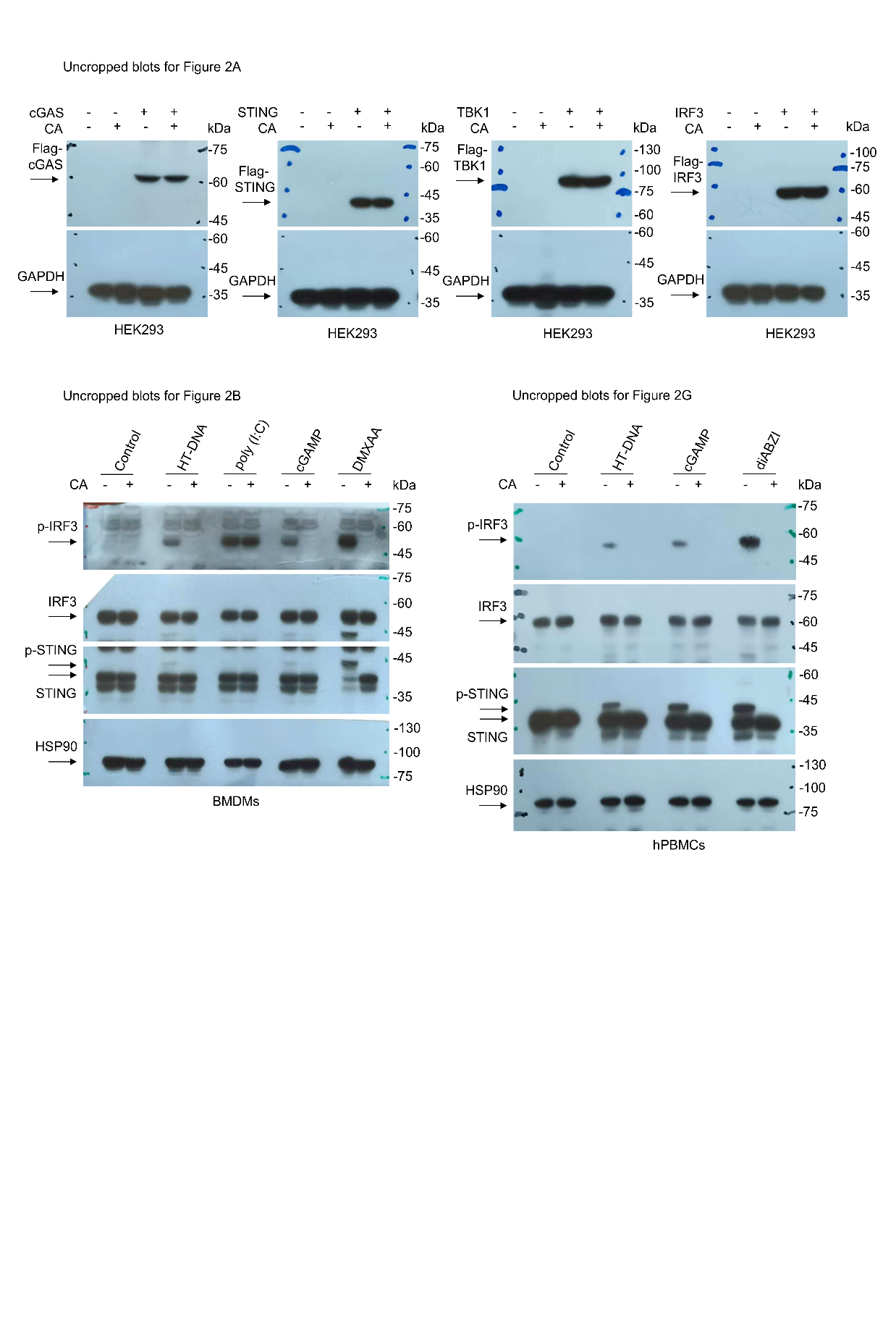


**Uncropped Blots for Figure 3**


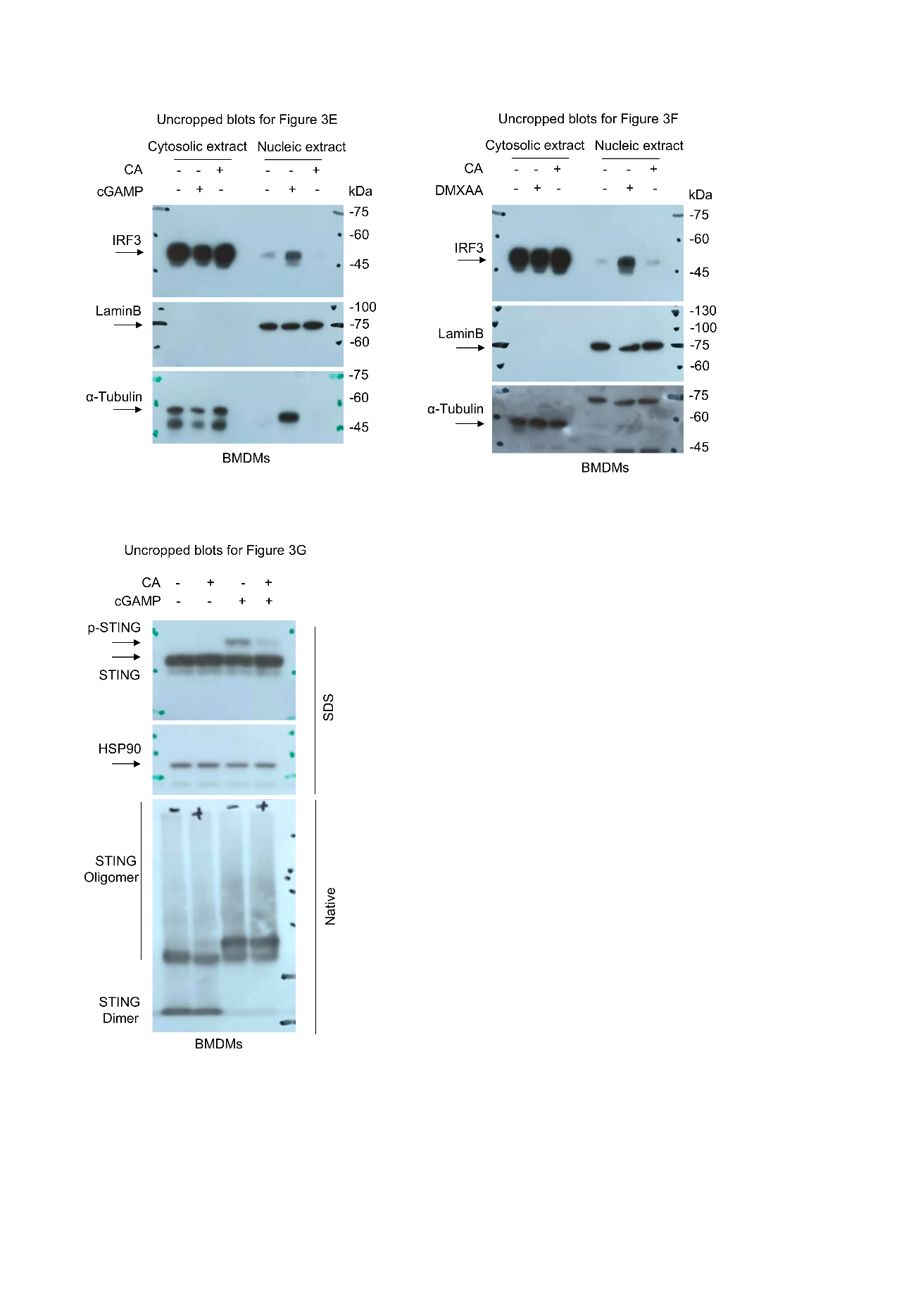


**Uncropped Blots for Figure 4**


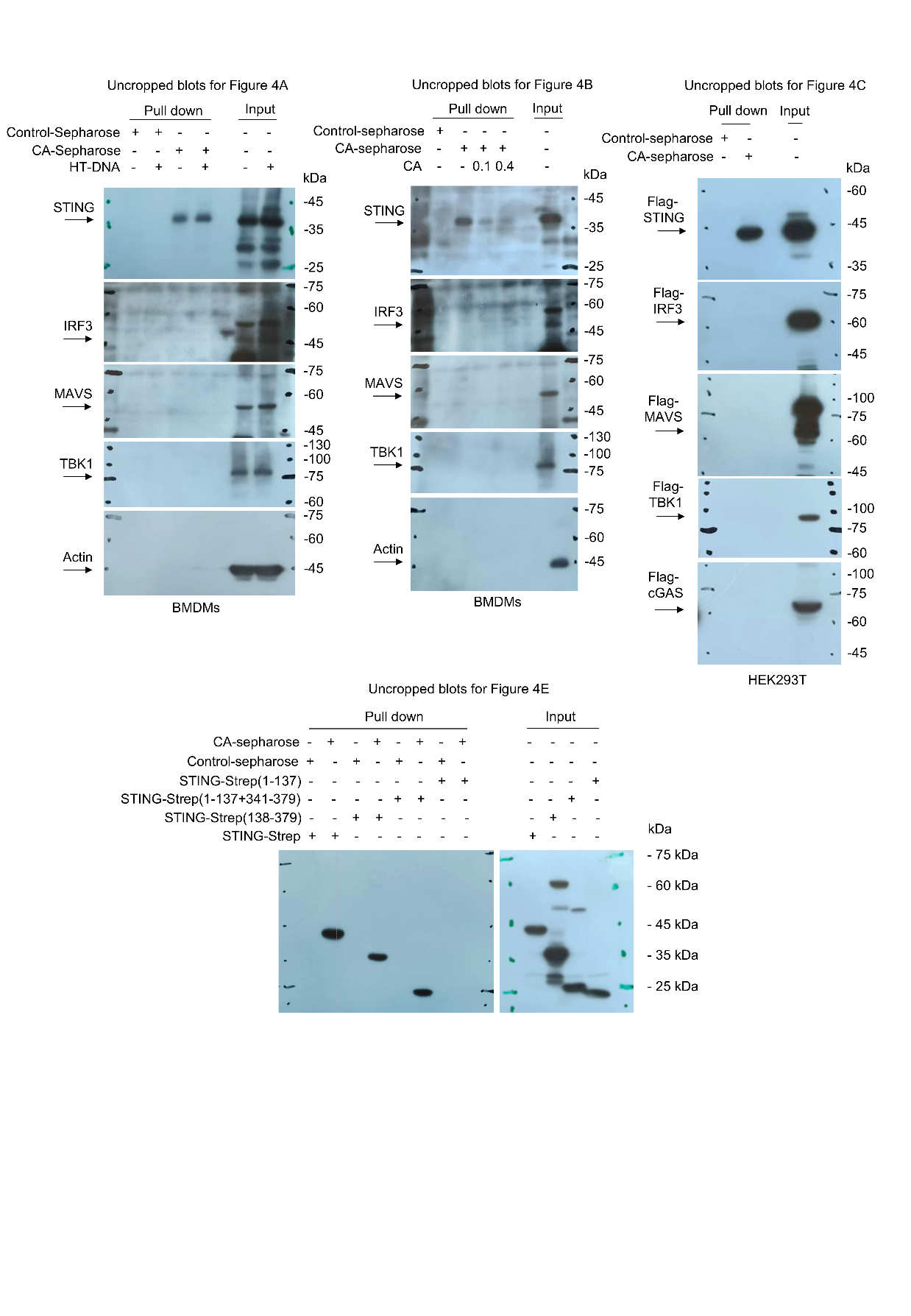


**Uncropped Blots for Figure 4**


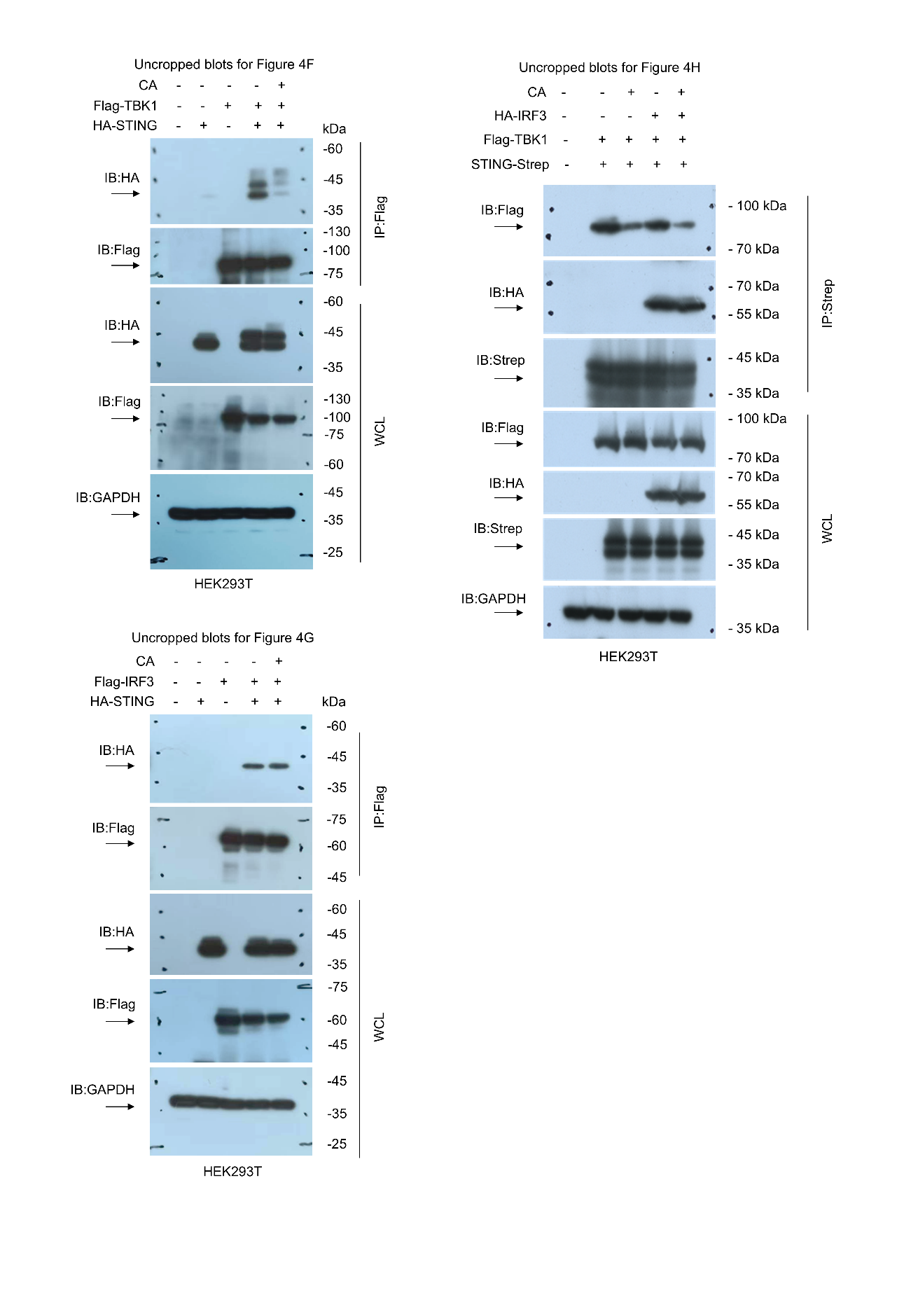


**Uncropped Blots for Figure 5**


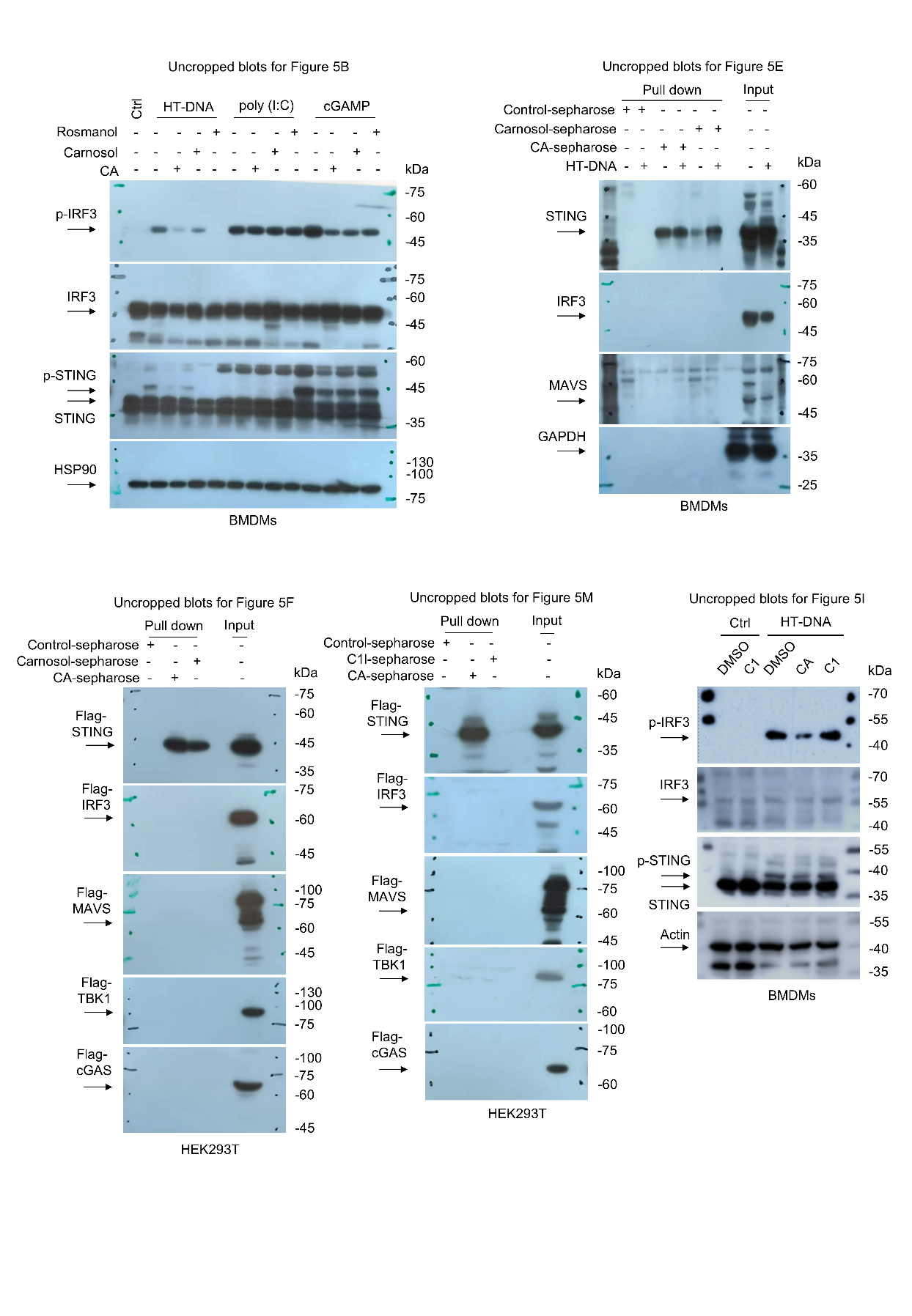


**Uncropped Blots for Figure S1**


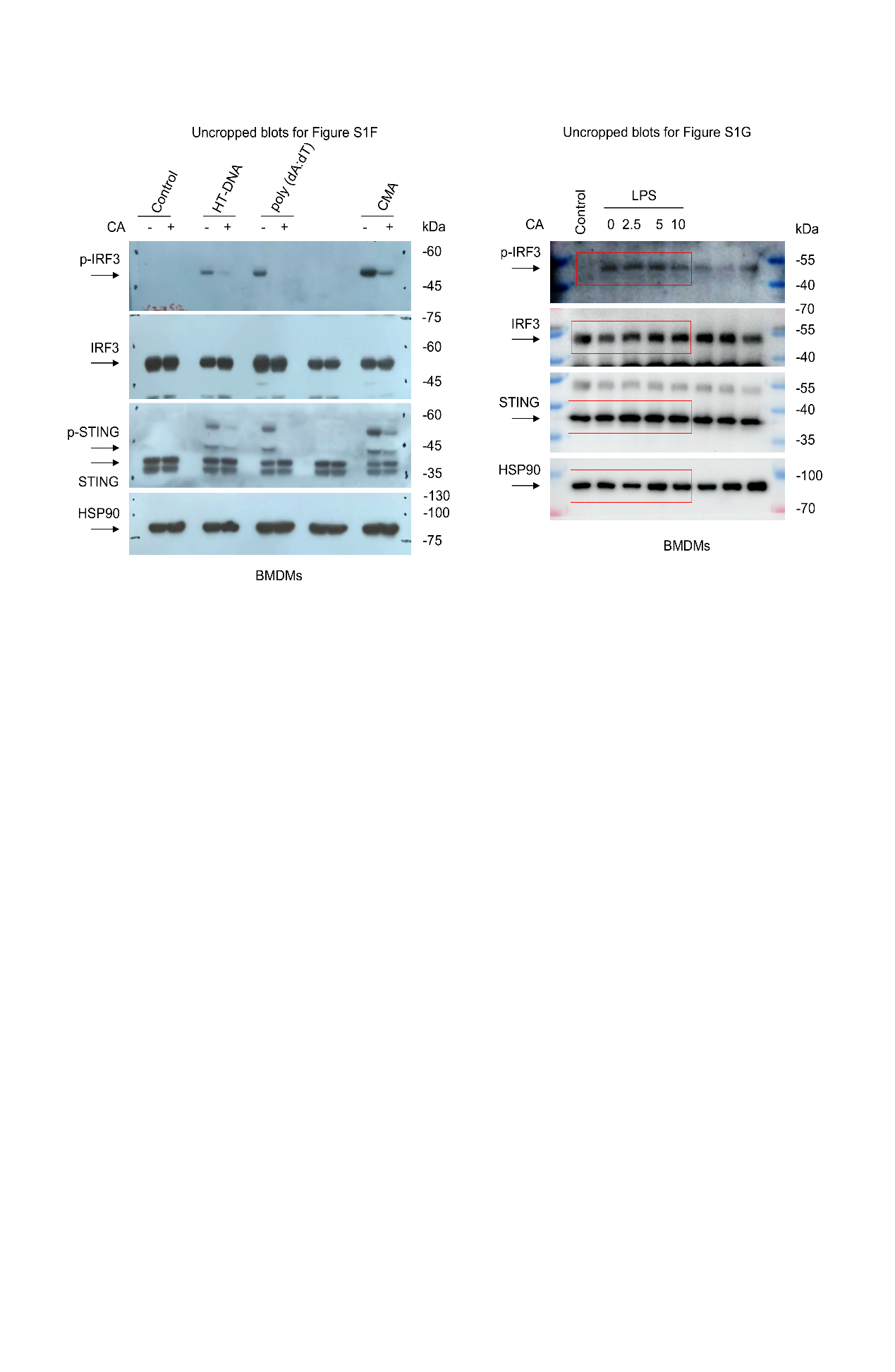


**Uncropped Blots for Figure S3 and S4**


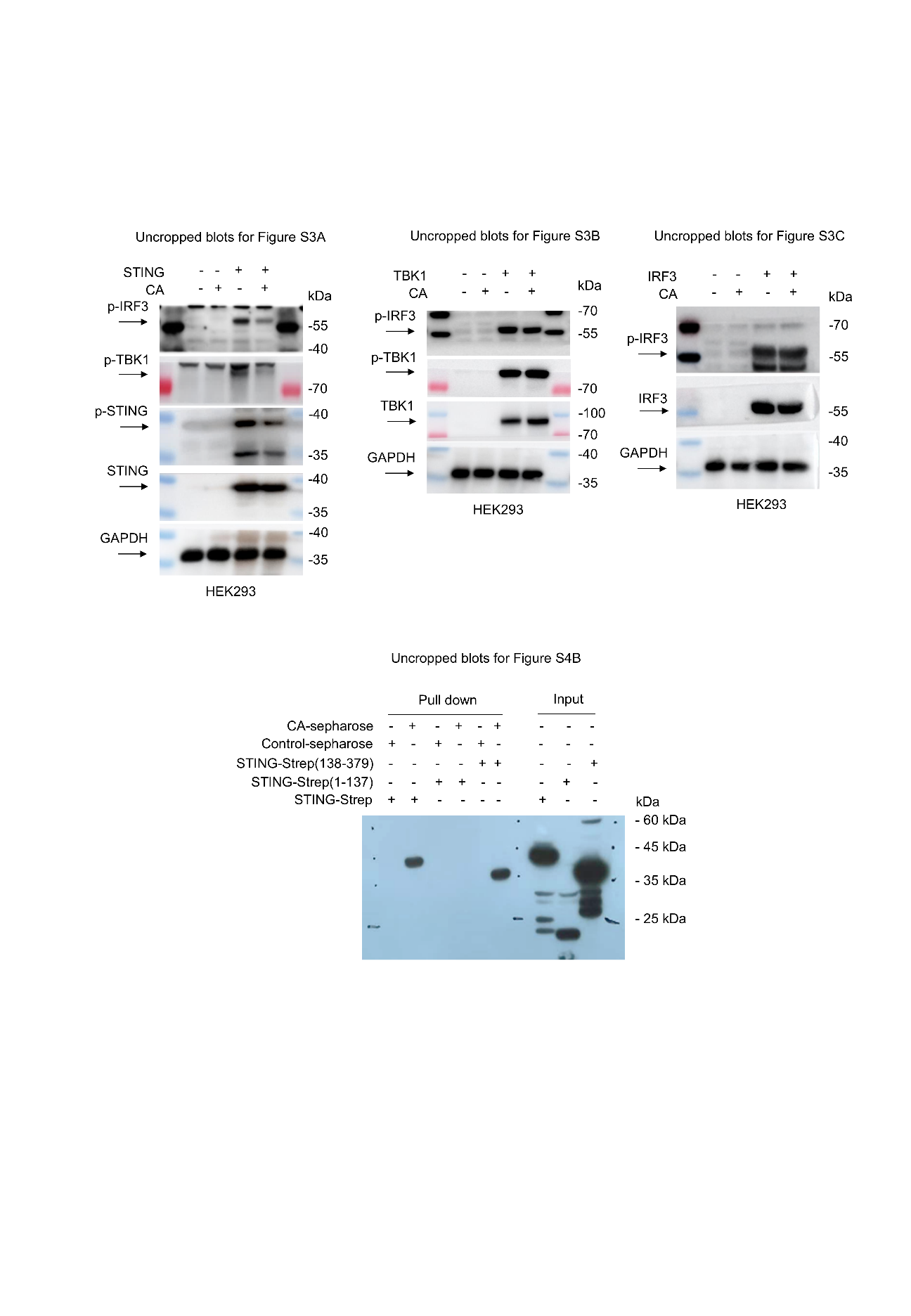


**Uncropped Blots for Figure S5 and S7**


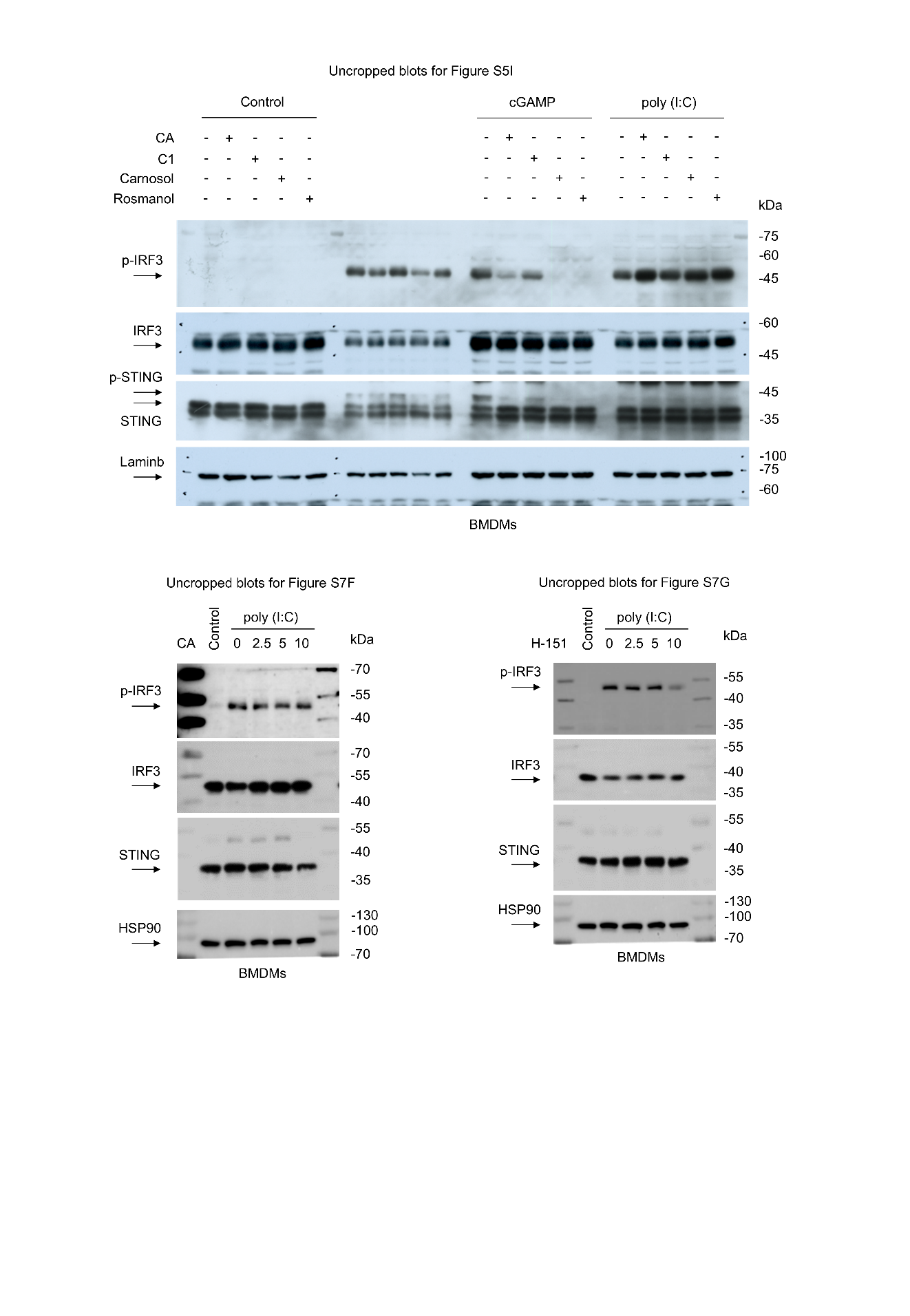

Supplement: Supplementary file 1 — Supporting Information [file ADVS-12-2417686-s001.docx]
